# CircTADA2A suppresses the progression of colorectal cancer via miR-374a-3p/KLF14 axis

**DOI:** 10.1186/s13046-020-01642-7

**Published:** 2020-08-15

**Authors:** Zhen Li, Hongyu Yao, Shihao Wang, Guobin Li, Xiaoming Gu

**Affiliations:** Department of Colorectal and Anal Surgery, the First Affiliated Hospital of Zhengzhou University, No.1 Jianshe East Road, Erqi District, Zhengzhou, 450052 Henan China

**Keywords:** Colorectal cancer, circTADA2A, miR-374a-3p, KLF14, Cell cycle, Apoptosis, Glycolysis

## Abstract

**Background:**

Colorectal cancer (CRC) is one of the causes of cancer-related death worldwide. The aim of our study was to disclose the expression pattern and underlying molecular mechanism of circular RNA TADA2A (circTADA2A) in CRC.

**Methods:**

The levels of circTADA2A, transcriptional adaptor 2A (TADA2A), microRNA-374a-3p (miR-374a-3p) and Kruppel like factor 14 (KLF14) were determined by quantitative real-time polymerase chain reaction (qRT-PCR). Xenograft tumor assay was used to uncover the function of circTADA2A in vivo. The miRNA targets of circTADA2A were searched using circbank and starbase softwares, while DIANA TOOL was used to explore miR-374a-3p-mRNA interactions. Dual-luciferase reporter assay and RNA immunoprecipitation (RIP) assay were performed to validate the target relationship of circTADA2A/miR-374a-3p/KLF14 axis. Cell cycle and apoptosis were analyzed by flow cytometry. The glycolysis of CRC cells was determined by Seahorse XF^e^ 96 Extracellular Flux Analyzer, Glucose Uptake Colorimetric Assay kit, Lactate Assay Kit II and ATP Colorimetric Assay kit. KLF14 protein level was measured by Western blot assay.

**Results:**

CircTADA2A was abnormally down-regulated in CRC tissues and cell lines. CircTADA2A overexpression impeded CRC tumor growth in vivo. MiR-374a-3p was verified as a target of circTADA2A in CRC cells, and circTADA2A inhibited the malignant potential of CRC cells through targeting miR-374a-3p. MiR-374a-3p interacted with KLF14 messenger RNA (mRNA), and miR-374a-3p deteriorated CRC through down-regulating KLF14. CircTADA2A enhanced the abundance of KLF14 through targeting miR-374a-3p in CRC cells.

**Conclusion:**

CircTADA2A functioned as a tumor suppressor in CRC to inhibit the glycolysis and cell cycle and potentiate the apoptosis of CRC cells via miR-374a-3p/KLF14 axis.

## Highlights

1. CircTADA2A overexpression represses the CRC tumor growth in vivo.
2. CircTADA2A inhibits the cell cycle, glycolytic metabolism and accelerates the apoptosis of CRC cells in vitro.
3. CircTADA2A could bind to miR-374a-3p, and KLF14 could directly interact with miR-374a-3p in CRC cells.
4. CircTADA2A exerts an anti-tumor role through up-regulating KLF14 via functioning as a ceRNA of miR-374a-3p in CRC.

## Background

Colorectal cancer (CRC) is one of the leading causes of cancer-associated death globally. The first-line treatment strategy for CRC remains surgery [1]. Although the therapeutic methods have been improved, the prognosis of patients with metastatic CRC is low [2]. Therefore, understanding the molecular mechanism behind the progression of CRC is crucial for CRC treatment.

Circular RNAs (circRNAs) are a special class of non-coding RNAs (ncRNAs) with covalently closed circular structure [3]. CircRNAs were found to be related to the pathogenesis of diseases [4]. Moreover, mounting articles have pointed out the crucial roles of circRNAs in cancers [5, 6]. For instance, Zhou et al. claimed that circRNA_0023642 accelerated the metastasis of gastric cancer cells through promoting EMT [7]. Han et al. proved that circMTO1 reduced the malignance of hepatocellular carcinoma cells through miR-9 [8]. Zeng et al. proved that circHIPK3 accelerated the proliferation and motility of CRC cells through miR-7 [9]. In this study, the expression profile and working mechanism of circTADA2A in CRC were explored.

MicroRNAs (miRNAs) refer to another class of ncRNAs, and they are 18–24 nucleotides in length [10]. CircRNAs have been found to serve as miRNAs sponges to regulate the levels of downstream proteins, and this mechanism was also called competitive endogenous RNA (ceRNA) mechanism [11]. For example, Zhang et al. claimed that circRNA_100269 blocked the progression of gastric cancer through targeting miR-630 [12]. Luan et al. found that circRNA_0084043 facilitated the development of malignant melanoma through targeting miR-153-3p [13]. The function of miR-374a-3p in cancers is still undefined. Herein, circTADA2A/miR-374a-3p axis was found in CRC, and the molecular mechanism was investigated.

Kruppel like factor 14 (KLF14) is a member of KLF protein family, and it serves as a transcription factor. Many members of KLF family have been reported to suppress the progression of cancers. For example, He et al. demonstrated that KLF6 hampered the proliferation of hepatocellular carcinoma cells [14]. Li et al. proved that KLF17 could inhibit the invasion of esophageal carcinoma cells [15]. Zhu et al. claimed that KLF4 suppressed the EMT and metastasis of pancreatic cancer cells [16]. The abundance of KLF14 has been reported to be down-regulated in CRC [17, 18]. Zhou et al. claimed that lncRNA HAND2-AS1 hampered the development of CRC through elevating the level of KLF14 via targeting miR-1275 [18]. Nevertheless, the potential regulatory mechanism behind KLF14-mediated influence on CRC cells remains to be revealed.

According to the data from Gene Expression Omnibus (GEO) database and clinical samples, we found that circTADA2A was down-regulated in CRC tissues and cells. The role of circTADA2A was explored in vivo and in vitro. The underlying mechanism of circTADA2A-mediated inhibition of CRC progression was then explored.

## Materials and methods

### Patients

Seventy pairs of CRC specimens and matching paracancerous specimens were obtained from CRC patients at the First Affiliated Hospital of Zhengzhou University. All tissues were stored in liquid nitrogen for the detection of circTADA2A, miR-374a-3p and KLF14. All tissue samples were pathologically diagnosed. The study was authorized by the Ethic Committee of the First Affiliated Hospital of Zhengzhou University. Written informed consent was provided from each subject.

### Cell culture

Human CRC cell lines (HCT116, SW620, LoVo and SW480) and normal human colon epithelial cell line NCM460 were obtained from BeNa Culture Collection (Beijing, China). These cell lines were maintained in Dulbecco’s Modified Eagle Medium (DMEM; Gibco, Carlsbad, CA, USA) supplemented with 10% fetal bovine serum (FBS; Gibco), 10% penicillin (100 U/mL) and 10% streptomycin (100 μg/mL) in an incubator with the environment of 37 °C and 5% CO_2_.

### Quantitative real-time polymerase chain reaction (qRT-PCR)

RNA was isolated with Trizol reagent (Life Technologies, Carlsbad, CA, USA). The purity and the concentration of different RNA samples were measured using NanoDrop ND-1000. The synthesis of complementary DNA (cDNA) was performed with Geneseed® II First Strand cDNA Synthesis Kit (Geneseed, Guangzhou, China) and All-in-OneTM miRNA First stand cDNA Synthesis Kit (GeneCopoeia, Rockville, MD, USA). Glyceraldehyde-3-phosphate dehydrogenase (GAPDH) and U6 served as the internal references in this study. The quantification of circTADA2A, transcriptional adaptor 2A (TADA2A) messenger RNA (mRNA), miR-374a-3p and KLF14 was carried out using the 2^−ΔΔCt^ method. The primers were shown as below: circTADA2A (Forward, 5′-TGTGCACCAAGACCAAGGAG-3′; Reverse, 5′-AGGAAAATCTGAAGTAGTGA-3′), TADA2A (Forward, 5′-CCTTTTTTCCTCTGCTTGCA-3′; Reverse, 5′-ATCCTGCCAATTTCCAAAGC-3′), miR-374a-3p (Forward, 5′-CUUAUCAGAUUGUAUUGUAAUU-3′; Reverse, 5′-AAUUACAAUACAAUCUGAUAAG-3′), KLF14 (Forward, 5′-TCGGAGGTGGGTGCGGCGCC-3′; Reverse, 5′-GGAGCCCTCGCCAGAGCTGC-3′), U6 (Forward, 5′-CTCGCTTCGGCAGCACA-3′; Reverse, 5′-AACGCTTCACGAATTTGCGT-3′), GAPDH (Forward, 5′-GGAGCCAAAAGGGTCATC-3′; Reverse, 5′-CCAGTGAGTTTCCCGTTC-3′), 18S rRNA (Forward, 5′-ACGGGCGCTGACCCCCCTTC-3′; Reverse, 5′-AGGGCAGACGTTCGAATGGG-3′).

### RNase R treatment and subcellular fractionation

Total RNA samples were treated with 3 U/mg RNase R (Epicentre Technologies, Madison, WI, USA) for 30 min at room temperature. qRT-PCR was employed to detect the levels of circTADA2A and TADA2A mRNA. Subcellular fractionation was conducted with PARIS™ Kit (Invitrogen, Carlsbad, CA, USA). 18S rRNA served as the cytoplasmic marker while U6 acted as nuclear marker in this study.

### Cell transfection

CircTADA2A overexpression plasmid (circTADA2A) and control empty vector (vector), KLF14 small interfering RNA (si-KLF14) and siRNA control (si-con), miR-374a-3p mimic (miR-374a-3p) and control (miR-con), miR-374a-3p inhibitor (anti-miR-374a-3p) and control (anti-miR-con) were purchased from Genepharma (Shanghai, China), and these plasmids or RNAs were transfected into CRC cells using Lipofectamine 3000 (Invitrogen).

### Xenograft tumor assay

BALB/c nude mice (5 weeks old) were purchased from Vital River Laboratory Animal Technology Co., Ltd. (Beijing, China) and randomly divided into 3 groups (*n* = 6). HCT116 or LoVo cells were stably transfected with vector or circTADA2A. The back region of BALB/c mice was subcutaneously injected with CRC cells stably expressing vector or circTADA2A or un-transfected CRC cells. The volume of tumors was measured every 7 d after inoculation for 7 d using a vernier caliper with the method of volume = π × (length × width^2^)/6. The mice were killed after inoculation for 35 d, and the tumors were weighed. Tumor tissues were subjected to measure the expression of circTADA2A by qRT-PCR. The procedures in this study were permitted by the Animal Research Committee of the First Affiliated Hospital of Zhengzhou University.

### Dual-luciferase reporter assay

The miR-374a-3p binding sequence in circTADA2A or the 3’untranslated region (3’UTR) of KLF14 was amplified and inserted to pGL3 luciferase reporter vector (Promega, Madison, WI, USA), and the recombinant luciferase reporter vector was termed as circTADA2A-wild-type (circTADA2A-WT) or KLF14-WT. The matching mutant type binding sites with miR-374a-3p in circTADA2A or the 3’UTR of KLF14 were also cloned to pGL3 luciferase reporter vector, generating circTADA2A-MUT or KLF14-MUT. LoVo and HCT116 cells were transfected with miR-con or miR-374a-3p and the above constructed reporter plasmids. The luciferase activities in different groups were determined by the dual-luciferase reporter assay system (Promega).

### RNA immunoprecipitation (RIP) assay

Magna RIP™ RNA-Binding Protein Immunoprecipitation kit (Millipore, Billerica, MA, USA) was used to conduct RIP assay. CRC cells were lysed with RIP buffer (Millipore). The cell lysate was incubated with sepharose beads (Bio-Rad, Hercules, CA, USA) pre-coated with Argonaute-2 antibody (Anti-Ago2). Immunoglobulin G antibody (Anti-IgG) served as the control in this study. qRT-PCR was applied to measure the abundance of circTADA2A, miR-374a-3p and KLF14 in the immunoprecipitated complexes.

### Cell cycle analysis by flow cytometry

CRC cells were harvested with cold phosphate buffer saline (PBS), and fixed in 70% cold ethanol solution (diluted with PBS) at − 20 °C overnight. After incubating with RNase (10 μM) for 30 min, propidine iodide (PI; 20 mg/mL; Solarbio, Beijing, China) was used to stain CRC cells for 20 min at 37 °C. The cell cycle was evaluated on a flow cytometer (BD Biosciences, San Jose, CA, USA).

### Cell apoptosis analysis by flow cytometry

CRC cells were collected and rinsed using cold PBS. Subsequently, CRC cells were suspended in Annexin-V binding buffer, and then these CRC cells were dyed using Annexin V combined fluorescein isothiocyanate (Annexin V-FITC; Solarbio) and PI (Solarbio) simultaneously in the dark for 15 min. The apoptotic CRC cells were tested on a flow cytometer (BD Biosciences).

### Glycolytic metabolism analysis

Seahorse XF^e^ 96 Extracellular Flux Analyzer (Seahorse Bioscience, Billerica, MA, USA) was used to detect the extracellular acidification rate (ECAR) and cellular oxygen consumption rate (OCR) using Seahorse XF Glycolysis Stress/Cell Mito Stress Test Kit (Seahorse Bioscience). For the detection of ECAR, Glucose, Oligomycin and 2-deoxyglucose (2-DG) were sequentially added to the wells of Seahorse XF 96 cell culture microplate. For the measure of OCR, Oligomycin, p-trifluoromethoxy carbonyl cyanide phenylhydrazone (FCCP) and the mitochondrial complex I inhibitor rotenone plus the mitochondrial complex III inhibitor antimycin A (Rote+AA) were sequentially added.

Glucose Uptake Colorimetric Assay kit (Biovision, Milpitas, California, USA), Lactate Assay Kit II (Biovision) and ATP Colorimetric Assay kit (Biovision) were utilized to detect the glucose consumption and the production of lactate and ATP in CRC cells according to the manufacturer’s instructions.

### Western blot assay

After relevant treatment, CRC cells were harvested using PBS and disrupted using Radioimmunoprecipitation assay (RIPA) solution (Beyotime, Shanghai, China). Protein samples (30 μg) were run on sodium dodecyl sulfate polyacrylamide gel electrophoresis (SDS-PAGE) gel and transferred to the polyvinylidene fluoride (PVDF) membrane. The PVDF membrane was blocked using 5% non-fat milk for 1 h, and then incubated with anti-KLF14 (ab85476; Abcam, Cambridge, MA, USA) or anti-β-actin (ab8226, Abcam) at 4 °C overnight. After washing with PBS-Tween 20 (PBST) for 3 times, the membrane was incubated with the secondary antibody (ab205718) for 2 h. After washing with PBST, the protein bands were measured by the enhanced chemiluminescent (ECL) system (Beyotime).

### Statistical analysis

GraphPad Prism 7.0 was used to analyze the data and generate the graphs. Student’s *t*-test or one-way analysis of variance (ANOVA) followed by Tukey’s test were used to calculate the *P* value between two groups or more than two groups. The correlation among the expression of miR-374a-3p, circTADA2A and KLF14 was analyzed using Spearman’s correlation coefficient. The differences between the enrichment of circTADA2A and the clinical characteristics of CRC patients were analyzed using χ^2^ test. *P* < 0.05 was identified as statistically significant.

## Results

### CircTADA2A is down-regulated in CRC tissues and cells

**Fig. 1:**
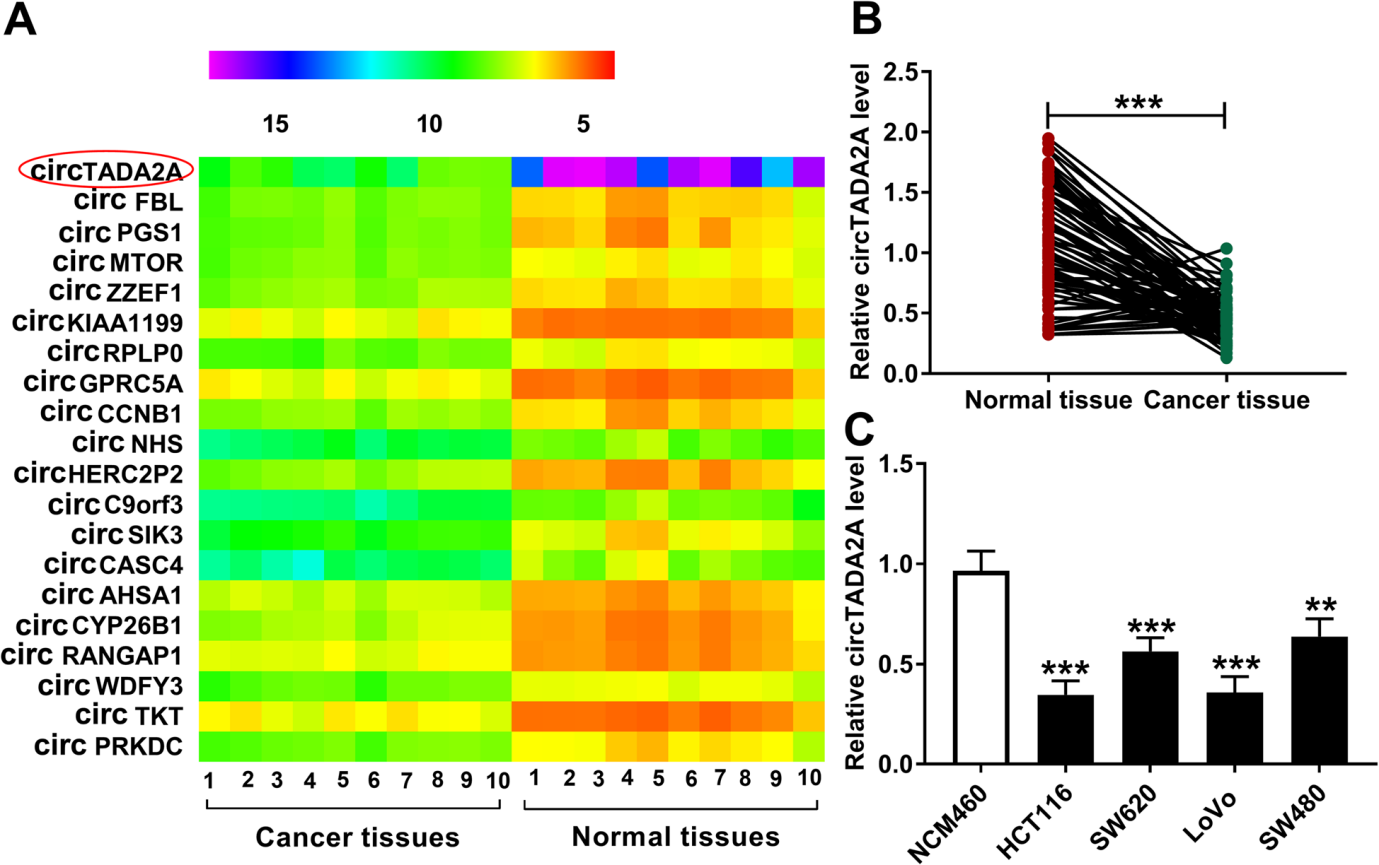
CircTADA2A is down-regulated in CRC tissues and cells. **a** The heat map revealed that the 20 most changed circRNAs in CRC tissues in comparison with matching normal tissues according to the GEO database (GSE126094). **b** The expression of circTADA2A was examined in CRC tissues (n = 70) and adjacent normal specimens (*n* = 70) by qRT-PCR. **c** The enrichment of circTADA2A was determined in normal human colon epithelial cell line NCM460 and four CRC cell lines by qRT-PCR. **P < 0.01, ***P < 0.001

According to the data of circRNA microarray from GEO database (GSE126094), we analyzed the expression patterns of circRNAs in 10 pairs of CRC tissues and non-tumor specimens. The top 20 differentially expressed circRNAs between CRC samples and adjacent normal samples were shown in Fig. 1a. The expression difference degree of these 20 circRNAs in CRC tissues and non-tumor specimens was listed in Table 1. Among these circRNAs, circTADA2A was the most down-regulated circRNA in CRC specimens (Table 1), thus circTADA2A was chosen for the following study. In addition, we also measured the expression of circTADA2A in a total of 70 pairs of CRC tissues and paracancerous tissues. A significant decrease in the expression of circTADA2A was observed in CRC tissues relative to normal tissues (Fig. 1b). The association between the expression of circTADA2A and the clinicopathologic features of CRC patients was shown in Table 2. CircTADA2A expression was negatively correlated with the tumor size, pT stage and distant metastasis of CRC tumors (Table 2). A panel of CRC cell lines, including HCT116, SW620, LoVo and SW480 and normal human colon epithelial cell line NCM460 were used to explore the expression profile of circTADA2A. As shown in Fig. 1c, circTADA2A was markedly down-regulated in CRC cells than that in NCM460 cells, especially in HCT116 and LoVo cells. Taken together, circTADA2A was notably down-regulated in CRC.

**Table 1:** The top 20 most significant circRNAs by differential expression analysis of GSE126094

SPOT_ID	adj. *P*.Val	*P*.Value	t	B	logFC	Gene symbol
hsa_circRNA_102049	3.59E-07	1.07E-10	−11.62095	14.65136	−6.44344	TADA2A
hsa_circRNA_102546	3.59E-07	2.68E-10	11.05463	13.76367	1.90394	FBL
hsa_circRNA_102211	3.59E-07	1.88E-10	11.27155	14.1081	2.1746	PGS1
hsa_circRNA_100051	4.44E-07	1.39E-09	10.09019	12.16314	1.35429	MTOR
hsa_circRNA_101951	4.44E-07	1.12E-09	10.21694	12.38013	1.5229	ZZEF1
hsa_circRNA_101622	4.44E-07	8.17E-10	10.39633	12.68374	1.54141	KIAA1199
hsa_circRNA_101174	4.44E-07	6.20E-10	10.55657	12.95151	1.58114	RPLP0
hsa_circRNA_101017	4.44E-07	1.19E-09	10.17997	12.31705	1.59671	GPRC5A
hsa_circRNA_103862	4.44E-07	1.55E-09	10.03103	12.06117	1.71377	CCNB1
hsa_circRNA_104983	4.44E-07	1.52E-09	10.04284	12.08155	1.93483	NHS
hsa_circRNA_101458	4.44E-07	1.00E-09	10.27966	12.48674	1.95278	HERC2P2
hsa_circRNA_104833	4.44E-07	1.27E-09	10.1424	12.25277	2.24332	C9orf3
hsa_circRNA_100956	4.44E-07	8.93E-10	10.34488	12.59708	2.27914	SIK3
hsa_circRNA_101002	4.44E-07	1.11E-09	10.22198	12.38872	2.72327	CASC4
hsa_circRNA_101415	4.60E-07	1.81E-09	9.94175	11.90642	1.45961	AHSA1
hsa_circRNA_102760	4.60E-07	1.84E-09	9.93529	11.89517	1.86753	CYP26B1
hsa_circRNA_103239	4.87E-07	3.45E-09	9.58543	11.27839	1.31968	RANGAP1
hsa_circRNA_103686	4.87E-07	2.16E-09	9.84565	11.73867	1.41052	WDFY3
hsa_circRNA_103392	4.87E-07	3.18E-09	9.63058	11.3589	1.45321	TKT
hsa_circRNA_104619	4.87E-07	3.07E-09	9.64932	11.39223	1.66032	PRKDC

**Table 2:** Association between the expression of circTADA2A with clinicopathologic features in patients with colorectal cancer

Clinicopathologic features	Relative circTADA2A level		*P* value
	Low (%)	High (%)	
Age (years)			0.548
≥ 60	27 (62.8)	16 (37.2)	
< 60	15 (55.6)	12 (44.4)	
Gender			0.217
Male	30 (65.2)	16 (34.8)	
Female	12 (50.0)	12 (50.0)	
Tumor size (cm)			0.001
≥ 4	24 (82.8)	5 (17.2)	
< 4	18 (43.9)	23 (56.1)	
pT stage			0.015
I + II	16 (45.7)	19 (54.3)	
III + IV	26 (74.3)	9 (25.7)	
Distant metastasis			0.004
Absent	24 (49.0)	25 (51.0)	
Present	18 (85.7)	3 (14.3)	
Differentiation			0.6884
Well or moderate	25 (58.1)	18 (41.9)	
Poor	17 (63.0)	10 (27.0)	
Tumor location			0.1458
Right-side colon	31 (66.0)	16 (34.0)	
Left-side colon	11 (47.8)	12 (52.2)	

### CircTADA2A possesses the closed loop structure and it mainly locates in the cytoplasm of CRC cells

**Fig. 2:**
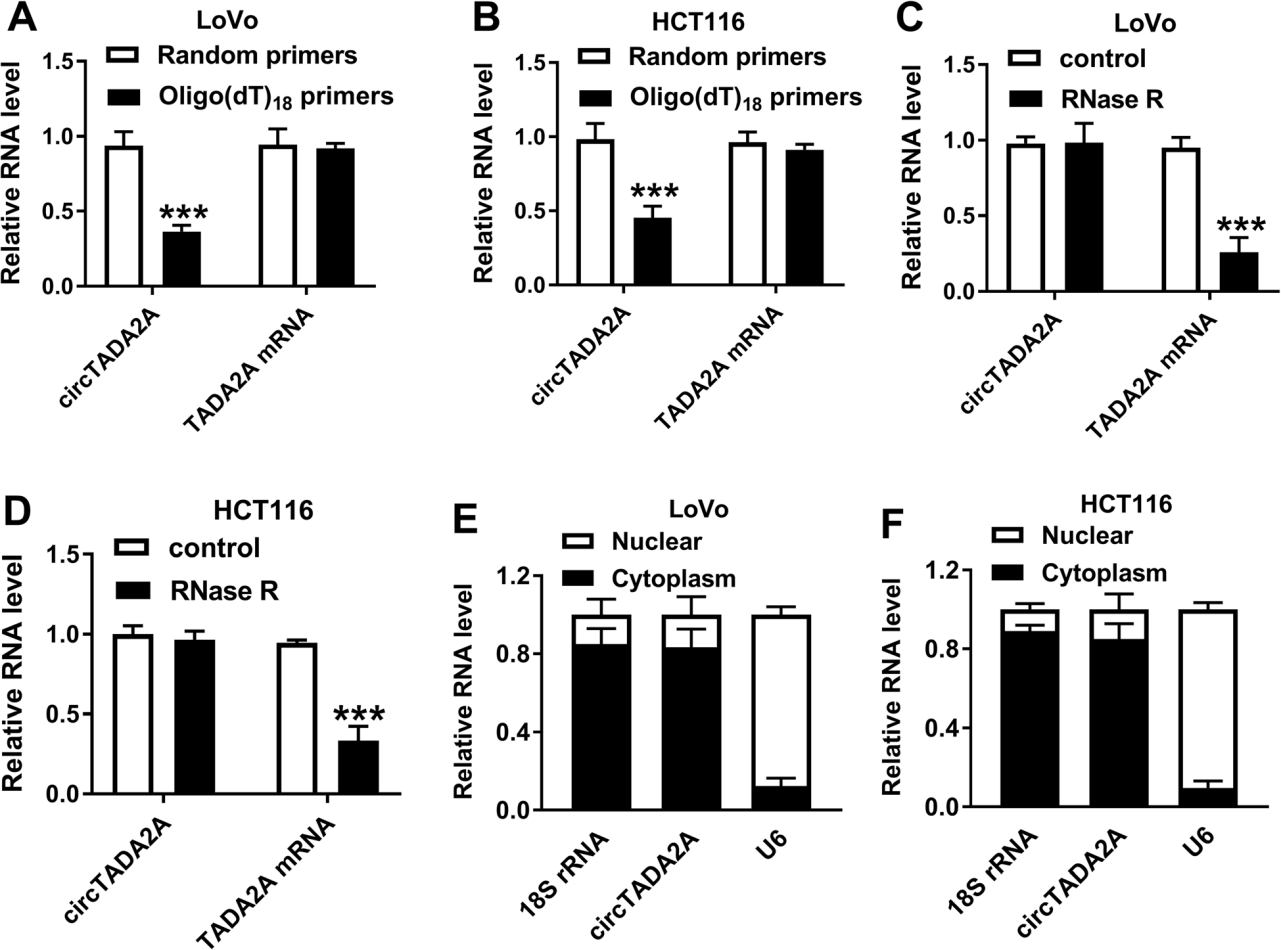
CircTADA2A possesses the closed loop structure and it mainly locates in the cytoplasm of CRC cells. **a** and **b** The relative expression of circTADA2A and TADA2A mRNA using random primers or oligo (dT)_18_ primers was measured by qRT-PCR. **c** and **d** The levels of circTADA2A and TADA2A mRNA were examined in control and RNase R treatment group by qRT-PCR. **e** and **f** The subcellular localization of circTADA2A was analyzed using PARIS™ Kit and qRT-PCR. ***P < 0.001

To test the circular structure and the stability of circTADA2A, Oligo (dT)_18_ primers and RNase R were used. As displayed in Fig. 2a and b, the level of circTADA2A was low in Oligo (dT)_18_ primers group relative to Random primers group, while the expression of its linear form remained unchanged in these two groups, suggesting that circTADA2A had no poly(A) tail. Besides, as exhibited in Fig. 2c and d, circTADA2A was resistant to RNase R compared with TADA2A mRNA. To explore the subcellular localization of circTADA2A, we isolated the nuclear fraction of CRC cells from the cytoplasmic fraction using PARIS™ Kit. CircTADA2A mainly distributed in the cytoplasm of CRC cells (Fig. 2e and f). These results revealed that circTADA2A possessed the circular structure and it mainly localized in the cytoplasm of CRC cells.

### CircTADA2A serves as a tumor suppressor in CRC in vivo

**Fig. 3:**
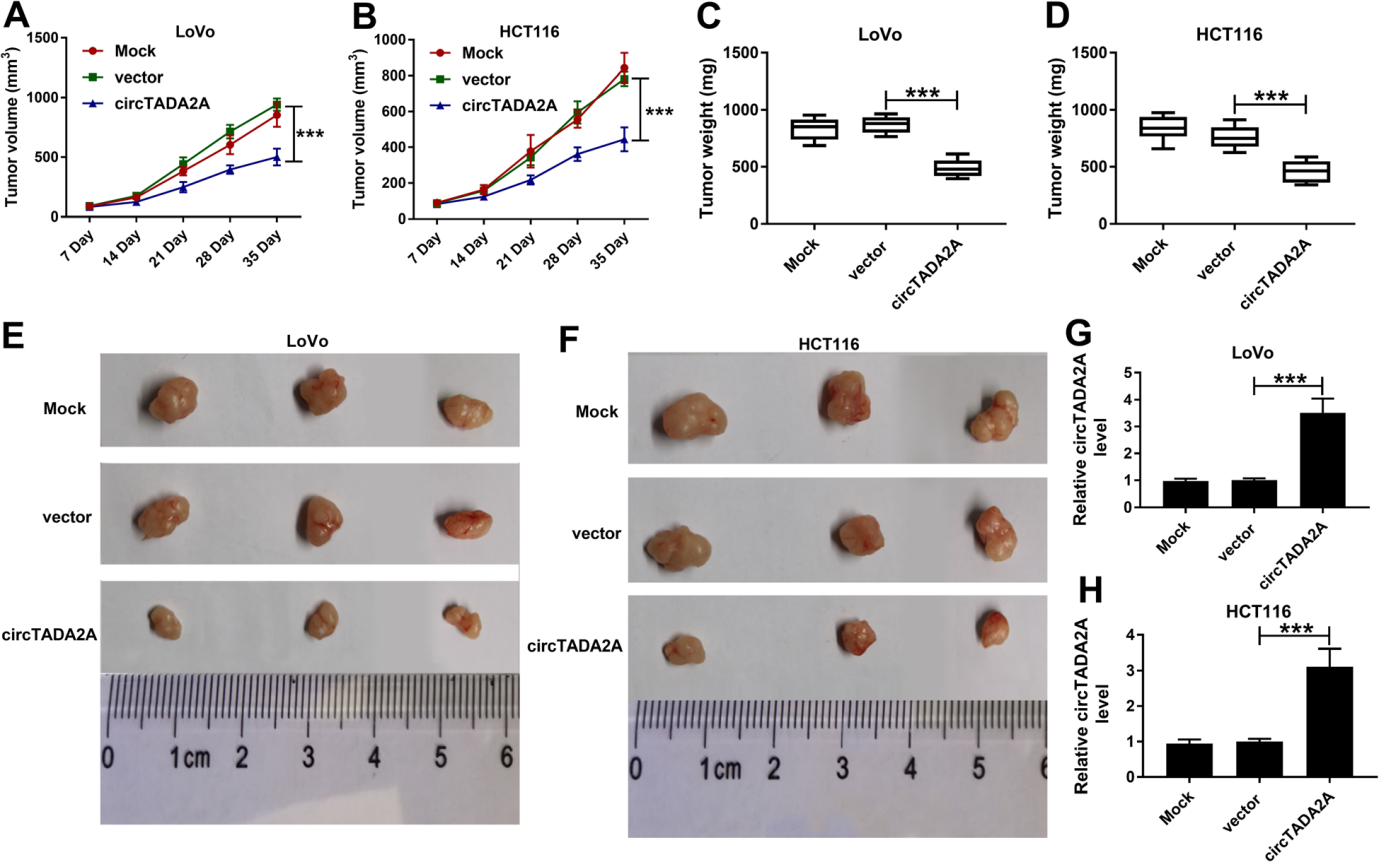
CircTADA2A serves as a tumor suppressor in CRC in vivo. Nude mice (*n* = 6) were subcutaneously injected with LoVo or HCT116 cells transfected with vector or circTADA2A. **a** and **b**) Tumor volume was analyzed every 7 d using the formula: volume = π × (length × width^2^)/6. **c** and **d** The tumors were weighted after dissecting from the nude mice at 35 d after injection. **e** and **f** Representative tumor diagrams in different groups were shown. **g** and **h** The level of circTADA2A was determined in tumor tissues from Mock group, vector group and circTADA2A group by qRT-PCR. ***P < 0.001

Xenograft tumor model was established to explore the function of circTADA2A in vivo. The mean volume and weight of CRC tumors derived from LoVo cells or HCT116 cells overexpressing circTADA2A were lower relative to Mock group or vector group (Fig. 3-3a-d). The representative tumor images in different groups were shown in Fig. 3e and f. The overexpression efficiency was also verified by qRT-PCR. There was a significant up-regulation in the abundance of circTADA2A in circTADA2A group than that in Mock or vector group (Fig. 3g and h). These findings indicated that circTADA2A suppressed the growth of CRC tumors in vivo.

### MiR-374a-3p is a target of circTADA2A

**Fig. 4:**
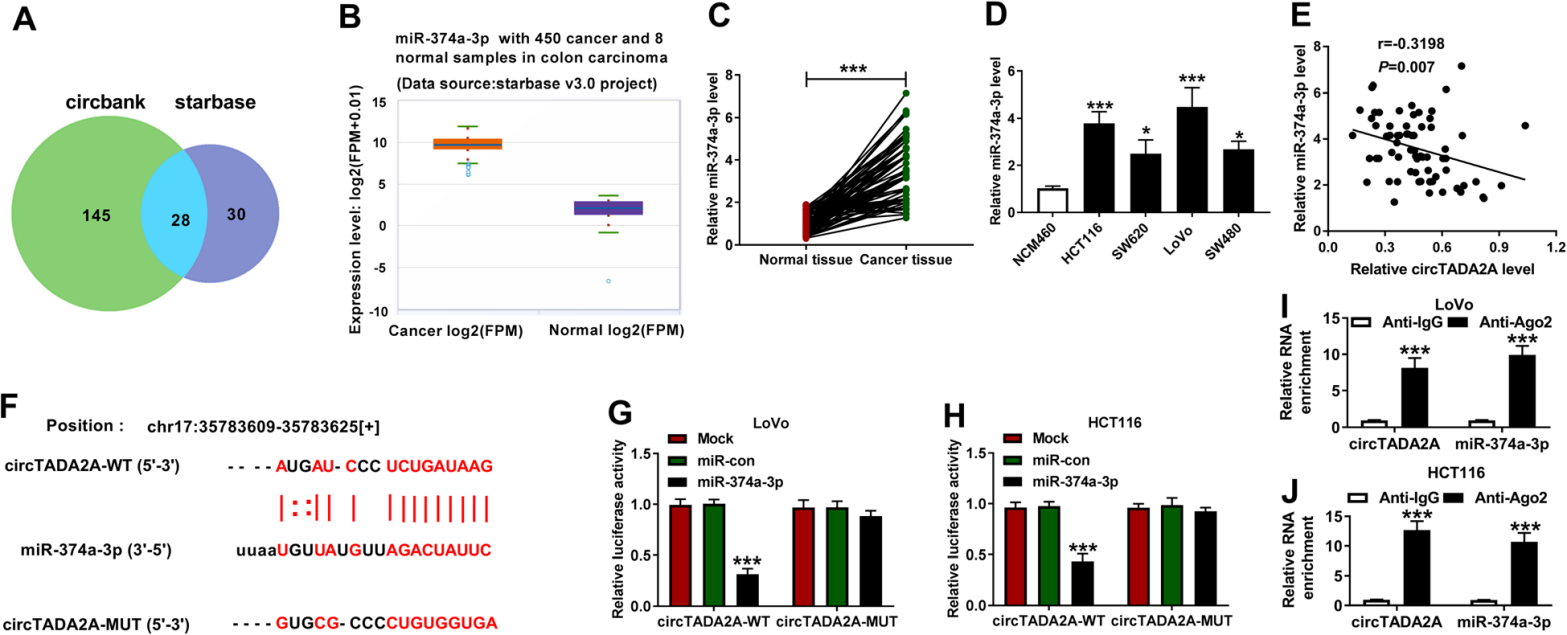
MiR-374a-3p is a target of circTADA2A. **a** A total of 28 candidate target genes of circTADA2A were simultaneously predicted by circbank and starbase softwares. **b** The expression of miR-374a-3p in colon carcinoma tissues (*n* = 450) and normal tissues (*n* = 8) from starbase v3.0 project was shown as a diagram. **c** The abundance of miR-374a-3p was examined in CRC tissue samples (*n* = 70) and normal tissue samples (*n* = 70) by qRT-PCR. **d** The expression of miR-374a-3p was determined in CRC cells and NCM460 cells by qRT-PCR. **e** The correlation analysis was conducted to assess the linear relationship between miR-374a-3p and circTADA2A in CRC tissues. **f** The predicted binding sequence between miR-374a-3p and circTADA2A and the mutant sites in circTADA2A were marked in red. **g** and **h**) Dual-luciferase reporter assay was performed to verify the interaction between miR-374a-3p and circTADA2A in LoVo and HCT116 cells. **i** and **j** RIP assay was conducted to test the combination between miR-374a-3p and circTADA2A in CRC cells. *P < 0.05, ***P < 0.001

Circbank and starbase softwares were utilized to seek the targets of circTADA2A (Sup Table 1 and Sup Table 2). As shown in Fig. 4a, there were 28 miRNAs that were predicted by both the two bioinformatic softwares. According to the starbase v3.0 project, among these 28 miRNAs, there existed 7 miRNAs that were highly expressed in CRC tissues in contrast to that in normal tissues, while a total of 11 miRNAs were found to be down-regulated in CRC tumor tissues relative to normal tissues (Table 3). In view of the low expression of circTADA2A in CRC tissues, we concentrated on these 7 highly expressed miRNAs. The function of miR-374a-3p had not been reported, so we focused on miR-374a-3p. As indicated in Fig. 4b, miR-374a-3p was abnormally up-regulated in colon carcinoma tissues (*n* = 450) relative to normal tissues (*n* = 8) according to the data from starbase v3.0 project. A marked up-regulation in the level of miR-374a-3p was also observed in CRC tissues (*n* = 70) compared with that in matching normal tissues (*n* = 70) (Fig. 4c). In addition, miR-374a-3p showed high expression in CRC cells than that in NCM460 cells (Fig. 4d). There was a negative correlation between the expression of circTADA2A and miR-374a-3p with the *P* value of 0.007 and r value of − 0.3198 (Fig. 4e). To confirm the target relationship between miR-374a-3p and circTADA2A, we mutated the binding sites with miR-374a-3p in circTADA2A (Fig. 4f). The sequence of circTADA2A containing wild-type or mutant type binding sites with miR-374a-3p was inserted into pGL3 luciferase reporter plasmid, generating circTADA2A-WT or circTADA2A-MUT. LoVo and HCT116 cells were co-transfected with miR-con or miR-374a-3p and the forementioned luciferase reporter plasmids. As indicated in Fig. 4g and h, the luciferase activity was dramatically reduced in circTADA2A-WT group when co-transfected with miR-374a-3p instead of miR-con, while the luciferase activity remained unaffected in circTADA2A-MUT group when co-transfected with miR-con or miR-374a-3p. Ago2 is an important component of RNA-induced silencing complex (RISC) that contained miRNAs, thus we tested if there was spatial target relationship between miR-374a-3p and circTADA2A in RISC via using Ago2 antibody (Anti-Ago2). CircTADA2A and miR-374a-3p were all enriched in Anti-Ago2 group compared with Anti-IgG group, suggested that miR-374a-3p bound to circTADA2A in CRC cells (Fig. 4i and j). Taken together, miR-374a-3p was a direct target of circTADA2A in CRC cells.

**Table 3:** A total of 28 candidate target genes of circTADA2A that were simultaneously predicted by circbank and starbase softwares

Low level	High level	No significant
hsa-let-7a-5p	hsa-miR-1294	hsa-miR-1321
hsa-let-7b-5p	hsa-miR-135a-5p	hsa-miR-221-3p
hsa-let-7c-5p	hsa-miR-135b-5p	hsa-miR-2467-3p
hsa-let-7d-5p	hsa-miR-193a-3p	hsa-miR-4262
hsa-let-7e-5p	hsa-miR-374a-3p	hsa-miR-4458
hsa-miR-181a-5p	hsa-miR-9-5p	hsa-miR-524-3p
hsa-miR-181b-5p	hsa-miR-98-5p	hsa-miR-525-3p
hsa-miR-193b-3p		hsa-miR-526b-5p
hsa-miR-214-3p		hsa-miR-6509-3p
hsa-miR-222-3p		hsa-miR-761
hsa-miR-942-5p		

### CircTADA2A mediates the inhibition in cell cycle and aerobic glycolysis and the acceleration in the apoptosis of CRC cells through targeting miR-374a-3p

**Fig. 5:**
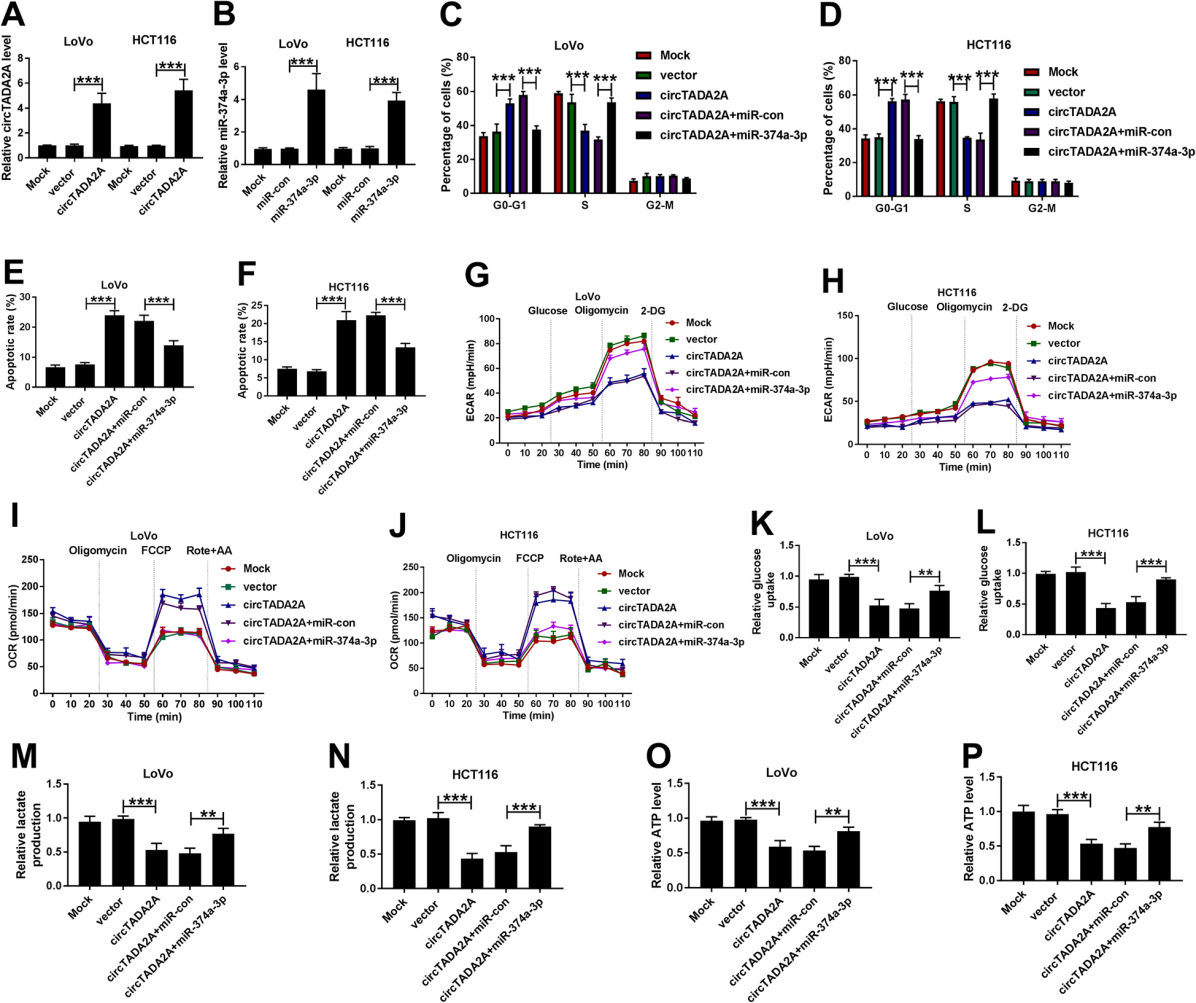
CircTADA2A mediates the inhibition in cell cycle and aerobic glycolysis and the acceleration in the apoptosis of CRC cells through targeting miR-374a-3p. **a** qRT-PCR was carried out to detect the level of circTADA2A in LoVo and HCT116 cells transfected with vector or circTADA2A. **b** qRT-PCR was employed to measure the enrichment of miR-374a-3p in LoVo and HCT116 cells transfected with miR-con or miR-374a-3p. **c-p** CRC cells were transfected with vector, circTADA2A, circTADA2A + miR-con or circTADA2A + miR-374a-3p, respectively. **c** and **d** The cell cycle of CRC cells was evaluated by flow cytometry. **e** and **f** The apoptosis of CRC cells was assessed by flow cytometry. **g-j** The ECAR and OCR of CRC cells were analyzed by Seahorse XF^e^ 96 Extracellular Flux Analyzer. **k-p** The glucose uptake, lactate production and ATP level in CRC cells were measured by Glucose Uptake Colorimetric Assay kit, Lactate Assay Kit II and ATP Colorimetric Assay kit, respectively. **P < 0.01, ***P < 0.001

To address whether circTADA2A exerted its role through targeting miR-374a-3p, we conducted rescue experiments. LoVo and HCT116 cells were transfected with the following four groups: vector, circTADA2A, circTADA2A + miR-con or circTADA2A + miR-374a-3p. The transfection efficiencies of circTADA2A and miR-374a-3p were both high in the two CRC cell lines (Fig. 5a and b). CircTADA2A overexpression elevated the percentage of CRC cells in G0-G1 stage, suggesting the inhibition of cell cycle in circTADA2A group, while the introduction of miR-374a-3p counteracted the inhibitory effect of circTADA2A accumulation on the cycle of CRC cells (Fig. 5c and d, Supplementary 1A and 1B). The apoptotic rate was notably increased in circTADA2A group, and the addition of miR-374a-3p impeded the apoptosis of CRC cells (Fig. 5e and f, Supplementary 1C and 1D). Warburg effect is one of the hallmarks of cancers, characterized by the promotion of glycolysis and the inhibition of the oxidative phosphorylation with the presence of oxygen [19]. We explored the influence of circTADA2A/miR-374a-3p axis on the Warburg effect of CRC cells through measuring the ECAR, OCR, glucose uptake, lactate production and ATP production. As exhibited in Fig. 5-5g-p, circTADA2A accumulation resulted in the inhibition of the ECAR, glucose uptake and the production of lactate and ATP and the promotion on the OCR of CRC cells, and these effects were attenuated by the co-transfection of circTADA2A and miR-374a-3p. These findings indicated that circTADA2A suppressed the cell cycle and glycolysis and promoted the apoptosis of CRC cells at least partly through targeting miR-374a-3p.

### MiR-374a-3p could bind to KLF14 in CRC cells

**Fig. 6:**
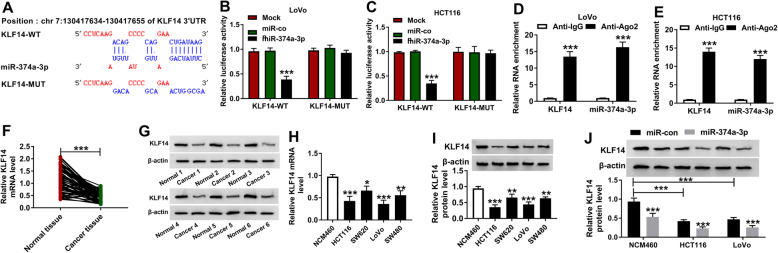
MiR-374a-3p could bind to KLF14 in CRC cells. **a** DIANA TOOL predicted that there existed binding sites between miR-374a-3p and KLF14 mRNA. **b** and **c** The luciferase activity was detected in CRC cells co-transfected with KLF14-WT or KLF14-MUT and miR-con or miR-374a-3p. **d** and **e** The target relationship between miR-374a-3p and KLF14 was also validated by RIP assay. **f** The mRNA level of KLF14 in CRC tissues (*n* = 70) and adjacent non-tumor tissues (*n* = 70) was determined by qRT-PCR. **g** The protein expression of KLF14 was examined in CRC tissues (*n* = 6) and matching normal tissues (*n* = 6) by Western blot assay. **h** and **i**) The abundance of KLF14 mRNA and protein was determined in four CRC cell lines and NCM460 cells by qRT-PCR and Western blot assay, respectively. *P < 0.05, **P < 0.01, ***P < 0.001

KLF14 was predicted as a target of miR-374a-3p by DIANA TOOL (Fig. 6a). The luciferase activity was decreased in KLF14-WT and miR-374a-3p co-transfected group other than miR-con and KLF14-WT co-transfected group, and there was no significant change in the luciferase activity of KLF14-MUT group with the addition of miR-con or miR-374a-3p (Fig. 6b and c). In addition, the levels of KLF14 and miR-374a-3p were high in the immunoprecipitated complex pulled-down by Ago2 antibody, suggested that KLF14 could bind to RNA induced silencing complex (RISC) (Fig. 6d and e). The enrichment of KLF14 mRNA and protein was notably reduced in CRC tissues relative to normal tissues (Fig. 6f and g). The mRNA and protein of KLF14 were also down-regulated in four CRC cell lines compared with NCM460 cells (Fig. 6h and i). MiR-374a-3p overexpression significantly down-regulated the protein expression of KLF14 in both CRC cell lines (HCT116 and LoVo) and NCM460 cell line (Fig. 6j). Collectively, KLF14 was a target of miR-374a-3p in CRC cells.

### KLF14 interference recovers the malignance of CRC cells which is restrained by the transfection of anti-miR-374a-3p

**Fig. 7:**
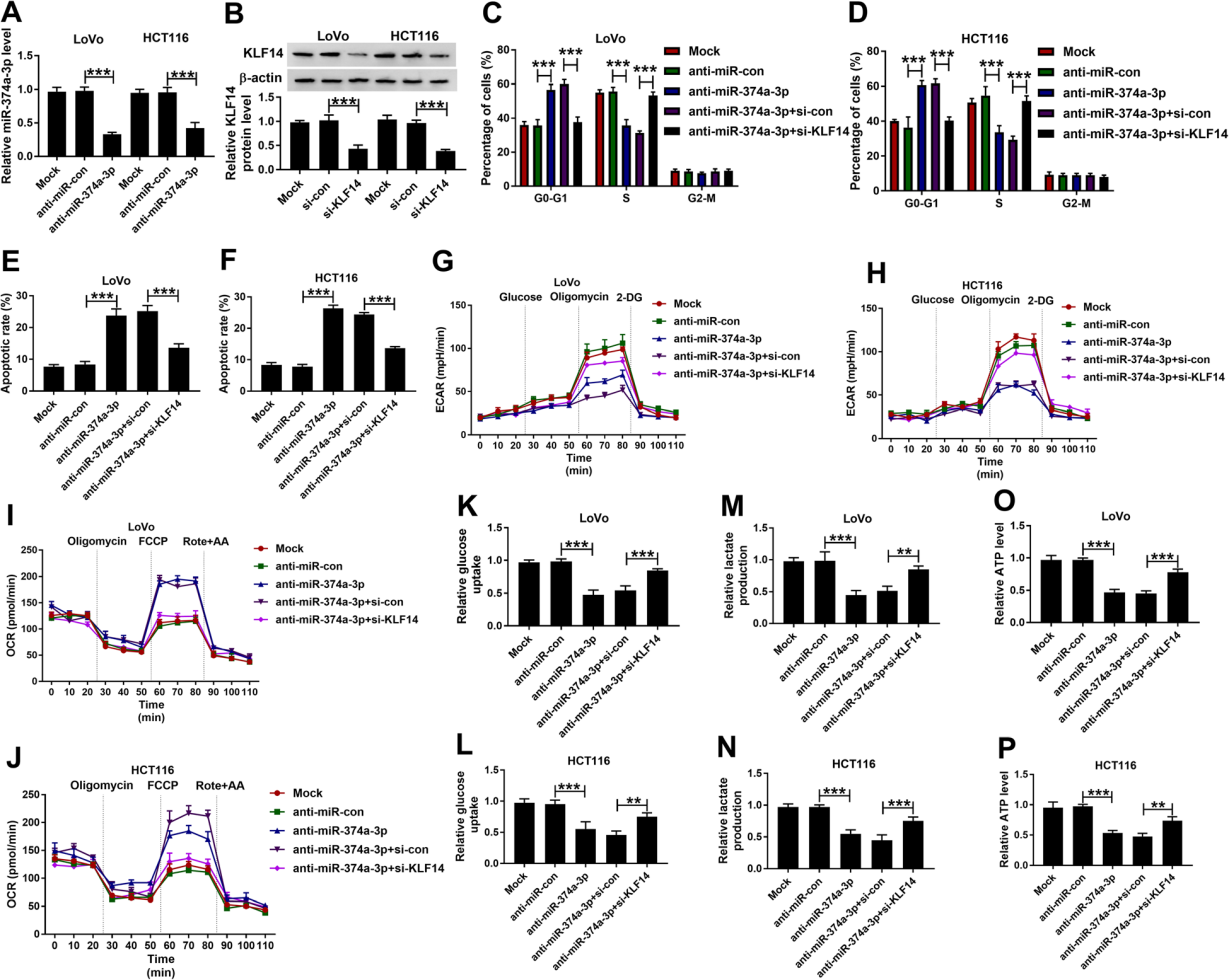
KLF14 interference recovers the malignance of CRC cells which is restrained by the transfection of anti-miR-374a-3p. **a** The level of miR-374a-3p was detected in CRC cells transfected with anti-miR-con or anti-miR-374a-3p by qRT-PCR. **b** Western blot assay was performed to detect the protein level of KLF14 in LoVo and HCT116 cells transfected with si-con or si-KLF14. **c-p** LoVo and HCT116 cells were transfected with anti-miR-con, anti-miR-374a-3p, anti-miR-374a-3p + si-con or anti-miR-374a-3p + si-KLF14. **c-f** Flow cytometry was applied to detect the cell cycle and apoptotic rate of CRC cells. **g-j** Seahorse XF^e^ 96 Extracellular Flux Analyzer was used to detect the ECAR and OCR of CRC cells. **k-p** Glucose Uptake Colorimetric Assay kit, Lactate Assay Kit II and ATP Colorimetric Assay kit were utilized to determine the glucose uptake and the production of lactate and ATP in CRC cells. **P < 0.01, ***P < 0.001

The transfection efficiencies of anti-miR-374a-3p and si-KLF14 were confirmed by qRT-PCR and Western blot. As shown in Fig. 7a, the transfection of anti-miR-374a-3p prominently reduced the abundance of miR-374a-3p in LoVo and HCT116 cells. The protein expression of KLF14 was notably declined in si-KLF14 group than that in si-con group (Fig. 7b). MiR-374a-3p expression was notably decreased with the transfection of anti-miR-374a-3p, and the addition of si-KLF14 did not affect the abundance of miR-374a-3p in CRC cells (Supplementary Fig. 2A and 2B). Besides, miR-374a-3p silencing up-regulated KLF14 expression in CRC cells, and the addition of si-KLF14 partly counteracted anti-miR-374a-3p-mediated promoting effect on KLF14 protein expression (Supplementary Fig. 2C and 2D). The cell cycle of CRC cells was blocked and the apoptosis was triggered with the depletion of miR-374a-3p, and the simultaneous transfection of anti-miR-374a-3p and si-KLF14 reversed the influence on the cell cycle and apoptosis caused by miR-374a-3p intervention (Fig. 7-7c and f). Furthermore, the interference of KLF14 also overturned the suppressive effects of miR-374a-3p silencing on the ECAR, glucose consumption, lactate production and ATP generation and the promoting effect on the OCR of CRC cells (Fig. 7-7g-p). Collectively, KLF14 depletion could partly reverse the biological effects of CRC cells mediated by miR-374a-3p intervention.

### The abundance of KLF14 is regulated by circTADA2A/miR-374a-3p axis in CRC cells

**Fig. 8:**
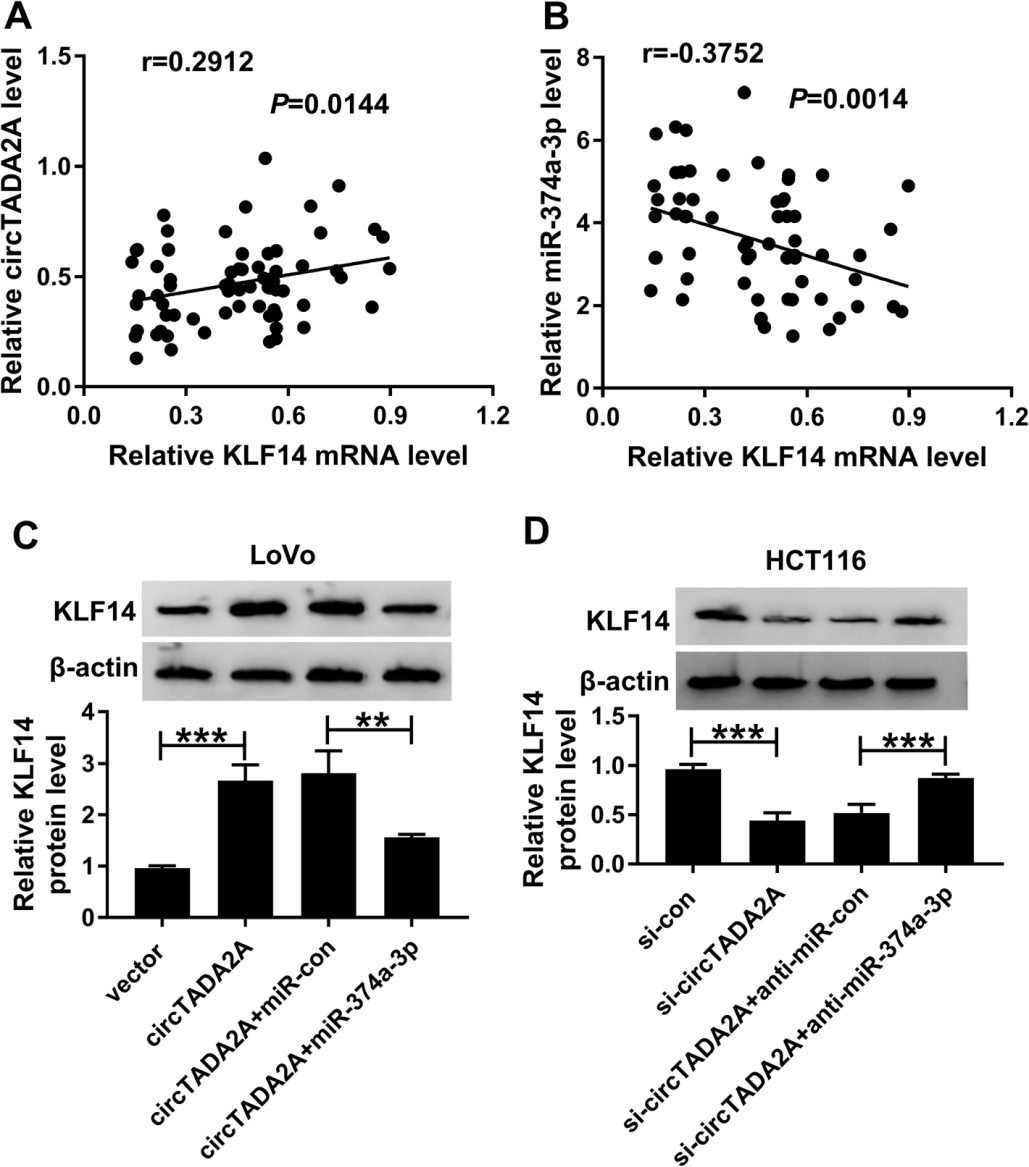
The abundance of KLF14 is regulated by circTADA2A/miR-374a-3p axis in CRC cells. **a** and **b** The linear relationship between the levels of KLF14 mRNA and circTADA2A or miR-374a-3p was analyzed through Spearman’s correlation coefficient. **c** The protein level of KLF14 was examined in LoVo cells transfected with vector, circTADA2A, circTADA2A + miR-con or circTADA2A + miR-374a-3p by Western blot assay. **d** Western blot assay was employed to detect the protein level of KLF14 in HCT116 cells transfected with si-con, si-circTADA2A, si-circTADA2A + anti-miR-con or si-circTADA2A + anti-miR-374a-3p. **P < 0.01, ***P < 0.001

We explored the linear relationship between KLF14 mRNA and circTADA2A or miR-374a-3p in CRC tissues (*n* = 70) through using Spearman’s correlation coefficient. KLF14 mRNA was positively correlated with circTADA2A and inversely correlated with miR-374a-3p (Fig. 8a and b). To clarify the relationship among KLF14, miR-374a-3p and circTADA2A, LoVo and HCT116 cells were transfected with the following four groups: vector, circTADA2A, circTADA2A + miR-con or circTADA2A + miR-374a-3p. The protein expression of KLF14 was up-regulated with the overexpression of circTADA2A, and the co-transfection of miR-374a-3p and circTADA2A declined the protein expression of KLF14 (Fig. 8c). Moreover, LoVo and HCT116 cells were co-transfected with si-circTADA2A and anti-miR-374a-3p. The depletion of miR-374a-3p recovered the protein abundance of KLF14, which was down-regulated by the transfection of si-circTADA2A (Fig. 8d). Based on these results, we concluded that circTADA2A could up-regulate KLF14 through functioning as a ceRNA of miR-374a-3p.

### CircTADA2A inhibits the tumor growth, cell cycle and glycolytic metabolism while promotes the apoptosis of CRC cells through up-regulating the expression of KLF14 via serving as a sponge for miR-374a-3p

**Fig. 9:**
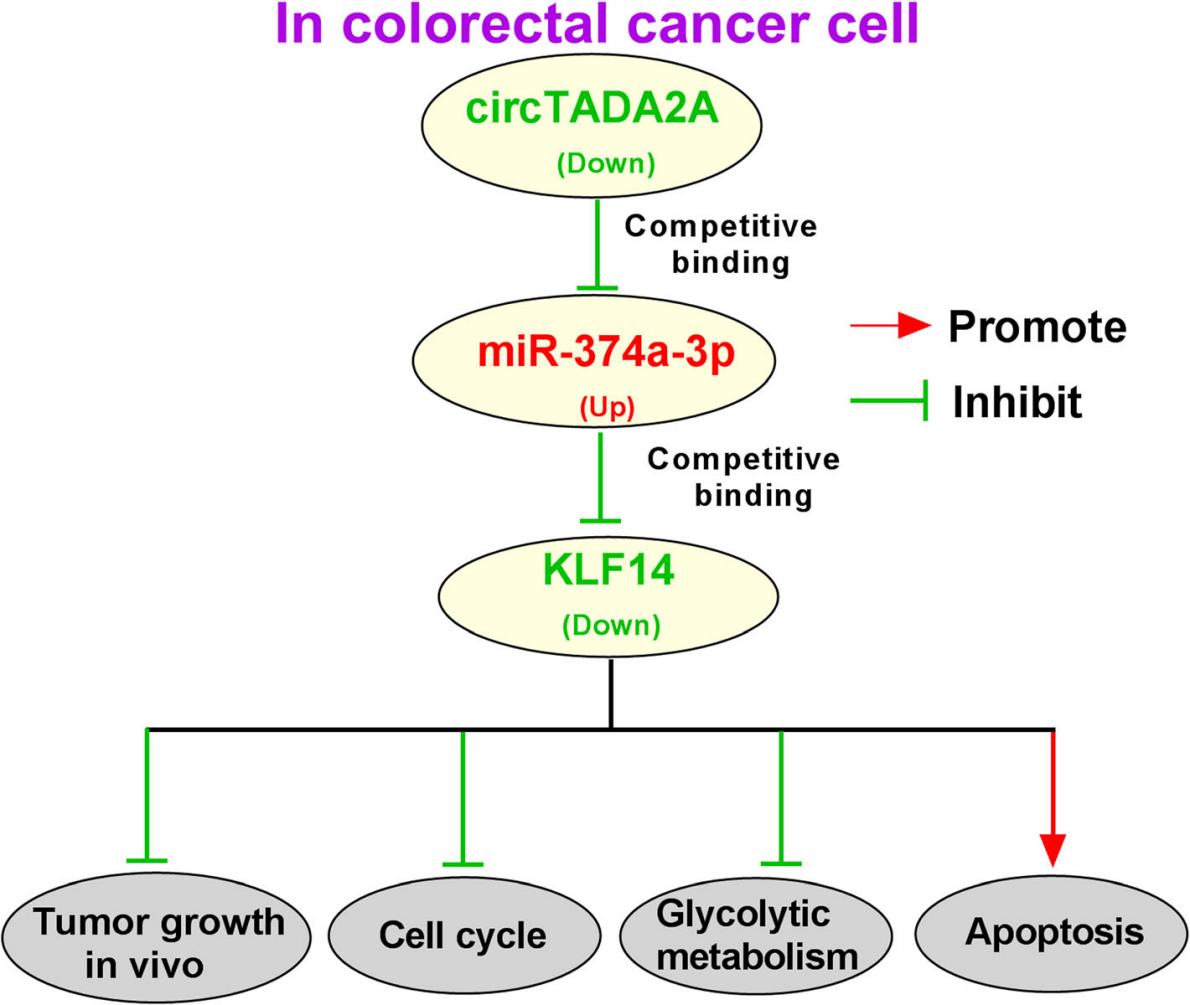
CircTADA2A inhibits the tumor growth, cell cycle and glycolytic metabolism while promotes the apoptosis of CRC cells through up-regulating the expression of KLF14 via serving as a sponge for miR-374a-3p

Based on the above findings, circTADA2A served as a tumor suppressor and it was down-regulated in CRC. The ectopic expression of circTADA2A could restrain the tumor growth, cell cycle and glycolysis and promote the apoptosis of CRC cells through miR-374a-3p/KLF14 axis (Fig. 9).

## Discussion

Investigating new diagnosis and prognosis biomarkers and uncovering the signal network behind the pathogenesis of CRC are essential for CRC treatment. In recent years, circRNAs have gotten a lot of attention. CircRNAs have been identified as novel biomarkers of cancers [5, 20]. For instance, Jiang et al. claimed that circRNA Cdr1as was a new prognostic marker of cholangiocarcinoma [21]. Yuan et al. revealed that circ_0026344 could serve as a prognostic marker for CRC, and circ_0026344 repressed the development of CRC through miR-21 and miR-31 [22]. The roles of circTADA2A in osteosarcoma and breast cancer have been reported. Wu et al. found that circTADA2A served as an oncogene in osteosarcoma, and circTADA2A accelerated the motility and proliferation of osteosarcoma cells via miR-203a-3p/CREB3 axis [23]. Xu et al. proved that circTADA2A prevented the proliferation and metastasis of breast cancer cells via miR-203a-3p/SOCS3 axis [24]. The diverse roles of circTADA2A in osteosarcoma and breast cancer might due to the heterogeneity of tumor microenvironment. However, the role of circTADA2A in CRC has never been reported. On the basis of the data from GEO dataset and 70 pairs CRC tissues and normal tissues, we found that circTADA2A was markedly down-regulated in CRC tissues relative to normal tissues. The level of circTADA2A was also found to be reduced in CRC cells compared with that in NCM460 cells. Murine xenograft model was established to test the role of circTADA2A in vivo. CircTADA2A overexpression inhibited the growth of CRC tumors in vivo, indicating the anti-tumor role of circTADA2A in CRC. Besides, circTADA2A overexpression also blocked the cell cycle and glycolysis and promoted the apoptosis of CRC cells in vitro.

MiR-374a-3p was found to be a target of circTADA2A in CRC cells through conducting dual-luciferase reporter assay and RIP assay. The function of miR-374a-3p has never been reported before. To explore if circTADA2A functioned through miR-374a-3p, we co-transfected circTADA2A and miR-374a-3p into LoVo and HCT116 cells. The addition of miR-374a-3p partly recovered the malignant potential of CRC cells which was suppressed by the accumulation of circTADA2A, demonstrated that circTADA2A suppressed the progression of CRC at least partly through targeting miR-374a-3p. MiR-374a-3p was one of the targets of circTADA2A, and its other miRNAs targets might also involved in circTADA2A-mediated influence in CRC. In further study, the signal network behind circTADA2A in CRC needs be further explored.

KLF14 has been reported to be a tumor suppressor in diverse cancers. Fan et al. reported that KLF14 depletion was associated with the amplification of centrosome and tumorigenesis [25]. Wang et al. demonstrated that lncRNA DGCR5 suppressed the progression of hepatocellular carcinoma through miR-346/KLF14 axis [26]. As for CRC, Zhou et al. found that KLF14 acted as the downstream gene of HAND2-AS1/miR-1275 axis to suppress the development of CRC [18]. Wu et al. claimed that KLF14 repressed the glycolysis of CRC cells through down-regulating glycolytic enzyme LDHB [17]. In this study, KLF14 was confirmed as a target of miR-374a-3p in CRC cells. Echoing with previous researches [17, 18], rescue experiments showed that KLF14 functioned as the target of miR-374a-3p to decline the malignance of CRC cells. Further experiments demonstrated that circTADA2A could enhance the expression of KLF14 through serving as a ceRNA for miR-374a-3p.

## Conclusion

Taken together, circTADA2A was found to be a tumor suppressor in CRC. CircTADA2A blocked the cell cycle, glycolysis and tumor growth while triggered the apoptosis of CRC cells through up-regulating KLF14 via targeting miR-374a-3p. These findings revealed that circTADA2A/miR-374a-3p/KLF14 axis could be a promising target and biomarker for CRC treatment.

## Supplementary information

**Additional file 1: Sup Table 1.** The prediction results of targets with circTADA2A from starbase.

**Additional file 2: Sup Table 2.** The prediction results of targets with circTADA2A from circbank.

**Additional file 3: Supplementary Figure 1.** The images of cell cycle and apoptosis in Fig. 5

**Additional file 4: Supplementary Figure 2.** The expression of miR-374a-3p and KLF14 in CRC cells under similar condition in Fig. 7**.** (A and B) The expression of miR-374a-3p was examined in CRC cells transfected with anti-miR-con, anti-miR-374a-3p, anti-miR-374a-3p + si-con or anti-miR-374a-3p + si-KLF14 by qRT-PCR. (C and D) Western blot assay was used to detect the protein level of KLF14 in CRC cells transfected with anti-miR-con, anti-miR-374a-3p, anti-miR-374a-3p + si-con or anti-miR-374a-3p + si-KLF14. ***P < 0.001.

## Data Availability

All remaining data are available within the article and supplementary files, or available from the authors upon request.

## Abbreviations

CRC: Colorectal cancer
circTADA2A: Circular RNA TADA2A
miR-374a-3p: microRNA-374a-3p
qRT-PCR: Quantitative real-time polymerase chain reaction
RIP: RNA immunoprecipitation
mRNA: Messenger RNA
circRNAs: Circular RNAs
miRNAs: MicroRNAs
ceRNA: Competitive endogenous RNA
KLF14: Kruppel like factor 14
GEO: Gene Expression Omnibus
