# Supplementary material for: CircTADA2A suppresses the progression of colorectal cancer via miR-374a-3p/KLF14 axis
Source: J Exp Clin Cancer Res. 2020 Aug 15;39:160. doi: 10.1186/s13046-020-01642-7 (PMC7429896; doi:10.1186/s13046-020-01642-7)
Supplement: Supplementary file 1 — Additional file 1: Sup Table 1. The prediction results of targets with circTADA2A from starbase. [file 13046_2020_1642_MOESM1_ESM.docx]

Sup Table 1. The prediction results of targets with circTADA2A from starbase

| miRNAid | miRNAname | geneID | geneName | geneType | chromosome | start | end |
| --- | --- | --- | --- | --- | --- | --- | --- |
| MIMAT0015069 | hsa-miR-3187-3p | NM_001488 | TADA2A | circRNA | chr17 | 35767331 | 35767357 |
| MIMAT0022844 | hsa-miR-216a-3p | NM_001488 | TADA2A | circRNA | chr17 | 35767339 | 35767362 |
| MIMAT0018109 | hsa-miR-3681-3p | NM_001488 | TADA2A | circRNA | chr17 | 35767342 | 35767362 |
| MIMAT0000424 | hsa-miR-128-3p | NM_001488 | TADA2A | circRNA | chr17 | 35767343 | 35767362 |
| MIMAT0026640 | hsa-miR-670-3p | NM_001166105 | TADA2A | circRNA | chr17 | 35771398 | 35771422 |
| MIMAT0026640 | hsa-miR-670-3p | NM_001488 | TADA2A | circRNA | chr17 | 35771398 | 35771422 |
| MIMAT0000441 | hsa-miR-9-5p | NM_001166105 | TADA2A | circRNA | chr17 | 35771406 | 35771430 |
| MIMAT0000441 | hsa-miR-9-5p | NM_001488 | TADA2A | circRNA | chr17 | 35771406 | 35771430 |
| MIMAT0002823 | hsa-miR-512-3p | NM_001166105 | TADA2A | circRNA | chr17 | 35771415 | 35771437 |
| MIMAT0002823 | hsa-miR-512-3p | NM_001488 | TADA2A | circRNA | chr17 | 35771415 | 35771437 |
| MIMAT0000703 | hsa-miR-361-5p | NM_001166105 | TADA2A | circRNA | chr17 | 35783609 | 35783624 |
| MIMAT0000703 | hsa-miR-361-5p | NM_001488 | TADA2A | circRNA | chr17 | 35783609 | 35783624 |
| MIMAT0004688 | hsa-miR-374a-3p | NM_001166105 | TADA2A | circRNA | chr17 | 35783609 | 35783625 |
| MIMAT0004688 | hsa-miR-374a-3p | NM_001488 | TADA2A | circRNA | chr17 | 35783609 | 35783625 |
| MIMAT0019908 | hsa-miR-4761-5p | NM_001166105 | TADA2A | circRNA | chr17 | 35783615 | 35783634 |
| MIMAT0019908 | hsa-miR-4761-5p | NM_001488 | TADA2A | circRNA | chr17 | 35783615 | 35783634 |
| MIMAT0000096 | hsa-miR-98-5p | NM_001166105 | TADA2A | circRNA | chr17 | 35783633 | 35783656 |
| MIMAT0000096 | hsa-miR-98-5p | NM_001488 | TADA2A | circRNA | chr17 | 35783633 | 35783656 |
| MIMAT0000062 | hsa-let-7a-5p | NM_001166105 | TADA2A | circRNA | chr17 | 35783635 | 35783656 |
| MIMAT0000062 | hsa-let-7a-5p | NM_001488 | TADA2A | circRNA | chr17 | 35783635 | 35783656 |
| MIMAT0000063 | hsa-let-7b-5p | NM_001166105 | TADA2A | circRNA | chr17 | 35783635 | 35783656 |
| MIMAT0000063 | hsa-let-7b-5p | NM_001488 | TADA2A | circRNA | chr17 | 35783635 | 35783656 |
| MIMAT0000064 | hsa-let-7c-5p | NM_001166105 | TADA2A | circRNA | chr17 | 35783635 | 35783656 |
| MIMAT0000064 | hsa-let-7c-5p | NM_001488 | TADA2A | circRNA | chr17 | 35783635 | 35783656 |
| MIMAT0000066 | hsa-let-7e-5p | NM_001166105 | TADA2A | circRNA | chr17 | 35783635 | 35783656 |
| MIMAT0000066 | hsa-let-7e-5p | NM_001488 | TADA2A | circRNA | chr17 | 35783635 | 35783656 |
| MIMAT0000067 | hsa-let-7f-5p | NM_001166105 | TADA2A | circRNA | chr17 | 35783635 | 35783656 |
| MIMAT0000067 | hsa-let-7f-5p | NM_001488 | TADA2A | circRNA | chr17 | 35783635 | 35783656 |
| MIMAT0000414 | hsa-let-7g-5p | NM_001166105 | TADA2A | circRNA | chr17 | 35783635 | 35783656 |
| MIMAT0000414 | hsa-let-7g-5p | NM_001488 | TADA2A | circRNA | chr17 | 35783635 | 35783656 |
| MIMAT0000415 | hsa-let-7i-5p | NM_001166105 | TADA2A | circRNA | chr17 | 35783635 | 35783656 |
| MIMAT0000415 | hsa-let-7i-5p | NM_001488 | TADA2A | circRNA | chr17 | 35783635 | 35783656 |
| MIMAT0000065 | hsa-let-7d-5p | NM_001166105 | TADA2A | circRNA | chr17 | 35783636 | 35783656 |
| MIMAT0000065 | hsa-let-7d-5p | NM_001488 | TADA2A | circRNA | chr17 | 35783636 | 35783656 |
| MIMAT0018980 | hsa-miR-4458 | NM_001166105 | TADA2A | circRNA | chr17 | 35783638 | 35783656 |
| MIMAT0018980 | hsa-miR-4458 | NM_001488 | TADA2A | circRNA | chr17 | 35783638 | 35783656 |
| MIMAT0019036 | hsa-miR-4500 | NM_001166105 | TADA2A | circRNA | chr17 | 35783640 | 35783656 |
| MIMAT0019036 | hsa-miR-4500 | NM_001488 | TADA2A | circRNA | chr17 | 35783640 | 35783656 |
| MIMAT0000256 | hsa-miR-181a-5p | NM_001166105 | TADA2A | circRNA | chr17 | 35783664 | 35783686 |
| MIMAT0000256 | hsa-miR-181a-5p | NM_001488 | TADA2A | circRNA | chr17 | 35783664 | 35783686 |
| MIMAT0000257 | hsa-miR-181b-5p | NM_001166105 | TADA2A | circRNA | chr17 | 35783664 | 35783686 |
| MIMAT0000257 | hsa-miR-181b-5p | NM_001488 | TADA2A | circRNA | chr17 | 35783664 | 35783686 |
| MIMAT0000258 | hsa-miR-181c-5p | NM_001166105 | TADA2A | circRNA | chr17 | 35783664 | 35783686 |
| MIMAT0000258 | hsa-miR-181c-5p | NM_001488 | TADA2A | circRNA | chr17 | 35783664 | 35783686 |
| MIMAT0002821 | hsa-miR-181d-5p | NM_001166105 | TADA2A | circRNA | chr17 | 35783664 | 35783686 |
| MIMAT0002821 | hsa-miR-181d-5p | NM_001488 | TADA2A | circRNA | chr17 | 35783664 | 35783686 |
| MIMAT0016894 | hsa-miR-4262 | NM_001166105 | TADA2A | circRNA | chr17 | 35783670 | 35783686 |
| MIMAT0016894 | hsa-miR-4262 | NM_001488 | TADA2A | circRNA | chr17 | 35783670 | 35783686 |
| MIMAT0019953 | hsa-miR-2467-3p | NM_001166105 | TADA2A | circRNA | chr17 | 35783689 | 35783710 |
| MIMAT0019953 | hsa-miR-2467-3p | NM_001488 | TADA2A | circRNA | chr17 | 35783689 | 35783710 |
| MIMAT0022706 | hsa-miR-561-5p | NM_001166105 | TADA2A | circRNA | chr17 | 35797841 | 35797862 |
| MIMAT0022706 | hsa-miR-561-5p | NM_001488 | TADA2A | circRNA | chr17 | 35797841 | 35797862 |
| MIMAT0004784 | hsa-miR-455-3p | NM_001166105 | TADA2A | circRNA | chr17 | 35797855 | 35797876 |
| MIMAT0004784 | hsa-miR-455-3p | NM_001488 | TADA2A | circRNA | chr17 | 35797855 | 35797876 |
| MIMAT0002835 | hsa-miR-526b-5p | NM_001166105 | TADA2A | circRNA | chr17 | 35797861 | 35797883 |
| MIMAT0002835 | hsa-miR-526b-5p | NM_001488 | TADA2A | circRNA | chr17 | 35797861 | 35797883 |
| MIMAT0004784 | hsa-miR-455-3p | NM_001166105 | TADA2A | circRNA | chr17 | 35797894 | 35797916 |
| MIMAT0004784 | hsa-miR-455-3p | NM_001488 | TADA2A | circRNA | chr17 | 35797894 | 35797916 |
| MIMAT0000278 | hsa-miR-221-3p | NM_001166105 | TADA2A | circRNA | chr17 | 35800606 | 35800618 |
| MIMAT0000278 | hsa-miR-221-3p | NM_001488 | TADA2A | circRNA | chr17 | 35800606 | 35800618 |
| MIMAT0000279 | hsa-miR-222-3p | NM_001166105 | TADA2A | circRNA | chr17 | 35800606 | 35800618 |
| MIMAT0000279 | hsa-miR-222-3p | NM_001488 | TADA2A | circRNA | chr17 | 35800606 | 35800618 |
| MIMAT0002173 | hsa-miR-483-3p | NM_001166105 | TADA2A | circRNA | chr17 | 35800631 | 35800651 |
| MIMAT0002173 | hsa-miR-483-3p | NM_001488 | TADA2A | circRNA | chr17 | 35800631 | 35800651 |
| MIMAT0000753 | hsa-miR-342-3p | NM_001166105 | TADA2A | circRNA | chr17 | 35800633 | 35800655 |
| MIMAT0000753 | hsa-miR-342-3p | NM_001488 | TADA2A | circRNA | chr17 | 35800633 | 35800655 |
| MIMAT0002830 | hsa-miR-520f-3p | NM_001166105 | TADA2A | circRNA | chr17 | 35800641 | 35800662 |
| MIMAT0002830 | hsa-miR-520f-3p | NM_001488 | TADA2A | circRNA | chr17 | 35800641 | 35800662 |
| MIMAT0000271 | hsa-miR-214-3p | NM_001166105 | TADA2A | circRNA | chr17 | 35800687 | 35800709 |
| MIMAT0000271 | hsa-miR-214-3p | NM_001488 | TADA2A | circRNA | chr17 | 35800687 | 35800709 |
| MIMAT0010364 | hsa-miR-761 | NM_001166105 | TADA2A | circRNA | chr17 | 35800687 | 35800709 |
| MIMAT0010364 | hsa-miR-761 | NM_001488 | TADA2A | circRNA | chr17 | 35800687 | 35800709 |
| MIMAT0017999 | hsa-miR-3619-5p | NM_001166105 | TADA2A | circRNA | chr17 | 35800691 | 35800709 |
| MIMAT0017999 | hsa-miR-3619-5p | NM_001488 | TADA2A | circRNA | chr17 | 35800691 | 35800709 |
| MIMAT0005789 | hsa-miR-513c-5p | NM_001488 | TADA2A | circRNA | chr17 | 35804810 | 35804831 |
| MIMAT0015087 | hsa-miR-514b-5p | NM_001488 | TADA2A | circRNA | chr17 | 35804810 | 35804831 |
| MIMAT0002806 | hsa-miR-490-3p | NM_001488 | TADA2A | circRNA | chr17 | 35818645 | 35818666 |
| MIMAT0026615 | hsa-miR-552-5p | NM_001488 | TADA2A | circRNA | chr17 | 35818649 | 35818669 |
| MIMAT0000459 | hsa-miR-193a-3p | NM_001488 | TADA2A | circRNA | chr17 | 35825592 | 35825614 |
| MIMAT0002819 | hsa-miR-193b-3p | NM_001488 | TADA2A | circRNA | chr17 | 35825593 | 35825614 |
| MIMAT0025475 | hsa-miR-6509-3p | NM_001488 | TADA2A | circRNA | chr17 | 35825598 | 35825617 |
| MIMAT0000428 | hsa-miR-135a-5p | NM_001488 | TADA2A | circRNA | chr17 | 35825620 | 35825642 |
| MIMAT0000758 | hsa-miR-135b-5p | NM_001488 | TADA2A | circRNA | chr17 | 35825620 | 35825642 |
| MIMAT0002835 | hsa-miR-526b-5p | NM_001488 | TADA2A | circRNA | chr17 | 35830507 | 35830531 |
| MIMAT0000262 | hsa-miR-187-3p | NM_001488 | TADA2A | circRNA | chr17 | 35830517 | 35830538 |
| MIMAT0002839 | hsa-miR-525-3p | NM_001488 | TADA2A | circRNA | chr17 | 35830532 | 35830554 |
| MIMAT0002850 | hsa-miR-524-3p | NM_001488 | TADA2A | circRNA | chr17 | 35830533 | 35830554 |
| MIMAT0028216 | hsa-miR-7153-5p | NM_001488 | TADA2A | circRNA | chr17 | 35830557 | 35830581 |
| MIMAT0002809 | hsa-miR-146b-5p | NM_001488 | TADA2A | circRNA | chr17 | 35830558 | 35830581 |
| MIMAT0000449 | hsa-miR-146a-5p | NM_001488 | TADA2A | circRNA | chr17 | 35830560 | 35830581 |
| MIMAT0005884 | hsa-miR-1294 | NM_001488 | TADA2A | circRNA | chr17 | 35834672 | 35834692 |
| MIMAT0019868 | hsa-miR-4739 | NM_001488 | TADA2A | circRNA | chr17 | 35834674 | 35834702 |
| MIMAT0019899 | hsa-miR-4756-5p | NM_001488 | TADA2A | circRNA | chr17 | 35834680 | 35834702 |
| MIMAT0005952 | hsa-miR-1321 | NM_001488 | TADA2A | circRNA | chr17 | 35834685 | 35834702 |
| MIMAT0004985 | hsa-miR-942-5p | NM_001488 | TADA2A | circRNA | chr17 | 35834693 | 35834714 |
