# Supplementary material for: CircTADA2A suppresses the progression of colorectal cancer via miR-374a-3p/KLF14 axis
Source: J Exp Clin Cancer Res. 2020 Aug 15;39:160. doi: 10.1186/s13046-020-01642-7 (PMC7429896; doi:10.1186/s13046-020-01642-7)
Supplement: Supplementary file 2 — Additional file 2: Sup Table 2. The prediction results of targets with circTADA2A from circbank. [file 13046_2020_1642_MOESM2_ESM.docx]

Sup Table 2. The prediction results of targets with circTADA2A from circbank

| circBank ID | circbase ID | length | miRNA ID (miR_ID) |
| --- | --- | --- | --- |
|  |  |  |  |
| hsa_circTADA2A_007 | hsa_circ_0106711 | 1798 | hsa-miR-578 |
| hsa_circTADA2A_007 | hsa_circ_0106711 | 1798 | hsa-miR-103a-2-5p |
| hsa_circTADA2A_007 | hsa_circ_0106711 | 1798 | hsa-miR-526b-5p |
| hsa_circTADA2A_007 | hsa_circ_0106711 | 1798 | hsa-miR-181a-5p |
| hsa_circTADA2A_007 | hsa_circ_0106711 | 1798 | hsa-miR-181b-5p |
| hsa_circTADA2A_007 | hsa_circ_0106711 | 1798 | hsa-miR-203a-3p |
| hsa_circTADA2A_007 | hsa_circ_0106711 | 1798 | hsa-miR-214-3p |
| hsa_circTADA2A_007 | hsa_circ_0106711 | 1798 | hsa-miR-2278 |
| hsa_circTADA2A_007 | hsa_circ_0106711 | 1798 | hsa-miR-3128 |
| hsa_circTADA2A_007 | hsa_circ_0106711 | 1798 | hsa-miR-365a-3p |
| hsa_circTADA2A_007 | hsa_circ_0106711 | 1798 | hsa-miR-365b-3p |
| hsa_circTADA2A_007 | hsa_circ_0106711 | 1798 | hsa-miR-4262 |
| hsa_circTADA2A_007 | hsa_circ_0106711 | 1798 | hsa-miR-4762-3p |
| hsa_circTADA2A_007 | hsa_circ_0106711 | 1798 | hsa-miR-5089-5p |
| hsa_circTADA2A_007 | hsa_circ_0106711 | 1798 | hsa-miR-513b-3p |
| hsa_circTADA2A_007 | hsa_circ_0106711 | 1798 | hsa-miR-5588-5p |
| hsa_circTADA2A_007 | hsa_circ_0106711 | 1798 | hsa-miR-6728-5p |
| hsa_circTADA2A_007 | hsa_circ_0106711 | 1798 | hsa-miR-6783-3p |
| hsa_circTADA2A_007 | hsa_circ_0106711 | 1798 | hsa-miR-6847-5p |
| hsa_circTADA2A_007 | hsa_circ_0106711 | 1798 | hsa-miR-6848-3p |
| hsa_circTADA2A_007 | hsa_circ_0106711 | 1798 | hsa-miR-6895-3p |
| hsa_circTADA2A_007 | hsa_circ_0106711 | 1798 | hsa-miR-761 |
| hsa_circTADA2A_007 | hsa_circ_0106711 | 1798 | hsa-miR-7854-3p |
| hsa_circTADA2A_007 | hsa_circ_0106711 | 1798 | hsa-miR-922 |
| hsa_circTADA2A_007 | hsa_circ_0106711 | 1798 | hsa-miR-942-5p |
| hsa_circTADA2A_007 | hsa_circ_0106711 | 1798 | hsa-let-7a-5p |
| hsa_circTADA2A_007 | hsa_circ_0106711 | 1798 | hsa-let-7b-5p |
| hsa_circTADA2A_007 | hsa_circ_0106711 | 1798 | hsa-let-7c-5p |
| hsa_circTADA2A_007 | hsa_circ_0106711 | 1798 | hsa-let-7d-5p |
| hsa_circTADA2A_007 | hsa_circ_0106711 | 1798 | hsa-let-7e-5p |
| hsa_circTADA2A_007 | hsa_circ_0106711 | 1798 | hsa-miR-1273g-3p |
| hsa_circTADA2A_007 | hsa_circ_0106711 | 1798 | hsa-miR-1294 |
| hsa_circTADA2A_007 | hsa_circ_0106711 | 1798 | hsa-miR-130a-5p |
| hsa_circTADA2A_007 | hsa_circ_0106711 | 1798 | hsa-miR-135a-5p |
| hsa_circTADA2A_007 | hsa_circ_0106711 | 1798 | hsa-miR-135b-5p |
| hsa_circTADA2A_007 | hsa_circ_0106711 | 1798 | hsa-miR-152-5p |
| hsa_circTADA2A_007 | hsa_circ_0106711 | 1798 | hsa-miR-2355-3p |
| hsa_circTADA2A_007 | hsa_circ_0106711 | 1798 | hsa-miR-2467-3p |
| hsa_circTADA2A_007 | hsa_circ_0106711 | 1798 | hsa-miR-3065-5p |
| hsa_circTADA2A_007 | hsa_circ_0106711 | 1798 | hsa-miR-3148 |
| hsa_circTADA2A_007 | hsa_circ_0106711 | 1798 | hsa-miR-335-3p |
| hsa_circTADA2A_007 | hsa_circ_0106711 | 1798 | hsa-miR-3692-5p |
| hsa_circTADA2A_007 | hsa_circ_0106711 | 1798 | hsa-miR-378a-3p |
| hsa_circTADA2A_007 | hsa_circ_0106711 | 1798 | hsa-miR-378c |
| hsa_circTADA2A_007 | hsa_circ_0106711 | 1798 | hsa-miR-378d |
| hsa_circTADA2A_007 | hsa_circ_0106711 | 1798 | hsa-miR-378h |
| hsa_circTADA2A_007 | hsa_circ_0106711 | 1798 | hsa-miR-422a |
| hsa_circTADA2A_007 | hsa_circ_0106711 | 1798 | hsa-miR-4270 |
| hsa_circTADA2A_007 | hsa_circ_0106711 | 1798 | hsa-miR-4303 |
| hsa_circTADA2A_007 | hsa_circ_0106711 | 1798 | hsa-miR-432-5p |
| hsa_circTADA2A_007 | hsa_circ_0106711 | 1798 | hsa-miR-4434 |
| hsa_circTADA2A_007 | hsa_circ_0106711 | 1798 | hsa-miR-4441 |
| hsa_circTADA2A_007 | hsa_circ_0106711 | 1798 | hsa-miR-4452 |
| hsa_circTADA2A_007 | hsa_circ_0106711 | 1798 | hsa-miR-4458 |
| hsa_circTADA2A_007 | hsa_circ_0106711 | 1798 | hsa-miR-4473 |
| hsa_circTADA2A_007 | hsa_circ_0106711 | 1798 | hsa-miR-452-3p |
| hsa_circTADA2A_007 | hsa_circ_0106711 | 1798 | hsa-miR-4531 |
| hsa_circTADA2A_007 | hsa_circ_0106711 | 1798 | hsa-miR-4659a-3p |
| hsa_circTADA2A_007 | hsa_circ_0106711 | 1798 | hsa-miR-4659b-3p |
| hsa_circTADA2A_007 | hsa_circ_0106711 | 1798 | hsa-miR-4755-5p |
| hsa_circTADA2A_007 | hsa_circ_0106711 | 1798 | hsa-miR-4765 |
| hsa_circTADA2A_007 | hsa_circ_0106711 | 1798 | hsa-miR-561-5p |
| hsa_circTADA2A_007 | hsa_circ_0106711 | 1798 | hsa-miR-5692b |
| hsa_circTADA2A_007 | hsa_circ_0106711 | 1798 | hsa-miR-5692c |
| hsa_circTADA2A_007 | hsa_circ_0106711 | 1798 | hsa-miR-6165 |
| hsa_circTADA2A_007 | hsa_circ_0106711 | 1798 | hsa-miR-619-5p |
| hsa_circTADA2A_007 | hsa_circ_0106711 | 1798 | hsa-miR-630 |
| hsa_circTADA2A_007 | hsa_circ_0106711 | 1798 | hsa-miR-6506-5p |
| hsa_circTADA2A_007 | hsa_circ_0106711 | 1798 | hsa-miR-6510-5p |
| hsa_circTADA2A_007 | hsa_circ_0106711 | 1798 | hsa-miR-6749-3p |
| hsa_circTADA2A_007 | hsa_circ_0106711 | 1798 | hsa-miR-6754-5p |
| hsa_circTADA2A_007 | hsa_circ_0106711 | 1798 | hsa-miR-6815-5p |
| hsa_circTADA2A_007 | hsa_circ_0106711 | 1798 | hsa-miR-6823-3p |
| hsa_circTADA2A_007 | hsa_circ_0106711 | 1798 | hsa-miR-6865-5p |
| hsa_circTADA2A_007 | hsa_circ_0106711 | 1798 | hsa-miR-6875-3p |
| hsa_circTADA2A_007 | hsa_circ_0106711 | 1798 | hsa-miR-6883-5p |
| hsa_circTADA2A_007 | hsa_circ_0106711 | 1798 | hsa-miR-6894-3p |
| hsa_circTADA2A_007 | hsa_circ_0106711 | 1798 | hsa-miR-7113-5p |
| hsa_circTADA2A_007 | hsa_circ_0106711 | 1798 | hsa-miR-7114-3p |
| hsa_circTADA2A_007 | hsa_circ_0106711 | 1798 | hsa-miR-889-5p |
| hsa_circTADA2A_007 | hsa_circ_0106711 | 1798 | hsa-miR-98-5p |
| hsa_circTADA2A_007 | hsa_circ_0106711 | 1798 | hsa-miR-103a-3p |
| hsa_circTADA2A_007 | hsa_circ_0106711 | 1798 | hsa-miR-107 |
| hsa_circTADA2A_007 | hsa_circ_0106711 | 1798 | hsa-miR-1178-5p |
| hsa_circTADA2A_007 | hsa_circ_0106711 | 1798 | hsa-miR-1236-3p |
| hsa_circTADA2A_007 | hsa_circ_0106711 | 1798 | hsa-miR-1253 |
| hsa_circTADA2A_007 | hsa_circ_0106711 | 1798 | hsa-miR-1279 |
| hsa_circTADA2A_007 | hsa_circ_0106711 | 1798 | hsa-miR-129-5p |
| hsa_circTADA2A_007 | hsa_circ_0106711 | 1798 | hsa-miR-1321 |
| hsa_circTADA2A_007 | hsa_circ_0106711 | 1798 | hsa-miR-141-3p |
| hsa_circTADA2A_007 | hsa_circ_0106711 | 1798 | hsa-miR-192-3p |
| hsa_circTADA2A_007 | hsa_circ_0106711 | 1798 | hsa-miR-193a-3p |
| hsa_circTADA2A_007 | hsa_circ_0106711 | 1798 | hsa-miR-193b-3p |
| hsa_circTADA2A_007 | hsa_circ_0106711 | 1798 | hsa-miR-197-5p |
| hsa_circTADA2A_007 | hsa_circ_0106711 | 1798 | hsa-miR-203a-5p |
| hsa_circTADA2A_007 | hsa_circ_0106711 | 1798 | hsa-miR-21-5p |
| hsa_circTADA2A_007 | hsa_circ_0106711 | 1798 | hsa-miR-218-2-3p |
| hsa_circTADA2A_007 | hsa_circ_0106711 | 1798 | hsa-miR-221-3p |
| hsa_circTADA2A_007 | hsa_circ_0106711 | 1798 | hsa-miR-222-3p |
| hsa_circTADA2A_007 | hsa_circ_0106711 | 1798 | hsa-miR-2276-3p |
| hsa_circTADA2A_007 | hsa_circ_0106711 | 1798 | hsa-miR-2392 |
| hsa_circTADA2A_007 | hsa_circ_0106711 | 1798 | hsa-miR-25-5p |
| hsa_circTADA2A_007 | hsa_circ_0106711 | 1798 | hsa-miR-302b-5p |
| hsa_circTADA2A_007 | hsa_circ_0106711 | 1798 | hsa-miR-302d-5p |
| hsa_circTADA2A_007 | hsa_circ_0106711 | 1798 | hsa-miR-3150b-3p |
| hsa_circTADA2A_007 | hsa_circ_0106711 | 1798 | hsa-miR-3151-3p |
| hsa_circTADA2A_007 | hsa_circ_0106711 | 1798 | hsa-miR-3152-3p |
| hsa_circTADA2A_007 | hsa_circ_0106711 | 1798 | hsa-miR-329-5p |
| hsa_circTADA2A_007 | hsa_circ_0106711 | 1798 | hsa-miR-3529-3p |
| hsa_circTADA2A_007 | hsa_circ_0106711 | 1798 | hsa-miR-3659 |
| hsa_circTADA2A_007 | hsa_circ_0106711 | 1798 | hsa-miR-3660 |
| hsa_circTADA2A_007 | hsa_circ_0106711 | 1798 | hsa-miR-3689d |
| hsa_circTADA2A_007 | hsa_circ_0106711 | 1798 | hsa-miR-374a-3p |
| hsa_circTADA2A_007 | hsa_circ_0106711 | 1798 | hsa-miR-378j |
| hsa_circTADA2A_007 | hsa_circ_0106711 | 1798 | hsa-miR-379-5p |
| hsa_circTADA2A_007 | hsa_circ_0106711 | 1798 | hsa-miR-3915 |
| hsa_circTADA2A_007 | hsa_circ_0106711 | 1798 | hsa-miR-3916 |
| hsa_circTADA2A_007 | hsa_circ_0106711 | 1798 | hsa-miR-3938 |
| hsa_circTADA2A_007 | hsa_circ_0106711 | 1798 | hsa-miR-411-5p |
| hsa_circTADA2A_007 | hsa_circ_0106711 | 1798 | hsa-miR-4252 |
| hsa_circTADA2A_007 | hsa_circ_0106711 | 1798 | hsa-miR-4448 |
| hsa_circTADA2A_007 | hsa_circ_0106711 | 1798 | hsa-miR-4475 |
| hsa_circTADA2A_007 | hsa_circ_0106711 | 1798 | hsa-miR-4494 |
| hsa_circTADA2A_007 | hsa_circ_0106711 | 1798 | hsa-miR-4495 |
| hsa_circTADA2A_007 | hsa_circ_0106711 | 1798 | hsa-miR-4509 |
| hsa_circTADA2A_007 | hsa_circ_0106711 | 1798 | hsa-miR-4533 |
| hsa_circTADA2A_007 | hsa_circ_0106711 | 1798 | hsa-miR-4534 |
| hsa_circTADA2A_007 | hsa_circ_0106711 | 1798 | hsa-miR-4535 |
| hsa_circTADA2A_007 | hsa_circ_0106711 | 1798 | hsa-miR-4653-3p |
| hsa_circTADA2A_007 | hsa_circ_0106711 | 1798 | hsa-miR-4679 |
| hsa_circTADA2A_007 | hsa_circ_0106711 | 1798 | hsa-miR-4680-3p |
| hsa_circTADA2A_007 | hsa_circ_0106711 | 1798 | hsa-miR-4686 |
| hsa_circTADA2A_007 | hsa_circ_0106711 | 1798 | hsa-miR-4711-3p |
| hsa_circTADA2A_007 | hsa_circ_0106711 | 1798 | hsa-miR-4742-3p |
| hsa_circTADA2A_007 | hsa_circ_0106711 | 1798 | hsa-miR-4761-5p |
| hsa_circTADA2A_007 | hsa_circ_0106711 | 1798 | hsa-miR-4775 |
| hsa_circTADA2A_007 | hsa_circ_0106711 | 1798 | hsa-miR-4793-5p |
| hsa_circTADA2A_007 | hsa_circ_0106711 | 1798 | hsa-miR-485-3p |
| hsa_circTADA2A_007 | hsa_circ_0106711 | 1798 | hsa-miR-5006-5p |
| hsa_circTADA2A_007 | hsa_circ_0106711 | 1798 | hsa-miR-5011-3p |
| hsa_circTADA2A_007 | hsa_circ_0106711 | 1798 | hsa-miR-509-5p |
| hsa_circTADA2A_007 | hsa_circ_0106711 | 1798 | hsa-miR-5096 |
| hsa_circTADA2A_007 | hsa_circ_0106711 | 1798 | hsa-miR-510-3p |
| hsa_circTADA2A_007 | hsa_circ_0106711 | 1798 | hsa-miR-510-5p |
| hsa_circTADA2A_007 | hsa_circ_0106711 | 1798 | hsa-miR-524-3p |
| hsa_circTADA2A_007 | hsa_circ_0106711 | 1798 | hsa-miR-525-3p |
| hsa_circTADA2A_007 | hsa_circ_0106711 | 1798 | hsa-miR-544a |
| hsa_circTADA2A_007 | hsa_circ_0106711 | 1798 | hsa-miR-5579-3p |
| hsa_circTADA2A_007 | hsa_circ_0106711 | 1798 | hsa-miR-5700 |
| hsa_circTADA2A_007 | hsa_circ_0106711 | 1798 | hsa-miR-585-5p |
| hsa_circTADA2A_007 | hsa_circ_0106711 | 1798 | hsa-miR-607 |
| hsa_circTADA2A_007 | hsa_circ_0106711 | 1798 | hsa-miR-6081 |
| hsa_circTADA2A_007 | hsa_circ_0106711 | 1798 | hsa-miR-619-3p |
| hsa_circTADA2A_007 | hsa_circ_0106711 | 1798 | hsa-miR-627-3p |
| hsa_circTADA2A_007 | hsa_circ_0106711 | 1798 | hsa-miR-6508-3p |
| hsa_circTADA2A_007 | hsa_circ_0106711 | 1798 | hsa-miR-6509-3p |
| hsa_circTADA2A_007 | hsa_circ_0106711 | 1798 | hsa-miR-6511a-3p |
| hsa_circTADA2A_007 | hsa_circ_0106711 | 1798 | hsa-miR-6755-5p |
| hsa_circTADA2A_007 | hsa_circ_0106711 | 1798 | hsa-miR-6777-5p |
| hsa_circTADA2A_007 | hsa_circ_0106711 | 1798 | hsa-miR-6780a-3p |
| hsa_circTADA2A_007 | hsa_circ_0106711 | 1798 | hsa-miR-6791-3p |
| hsa_circTADA2A_007 | hsa_circ_0106711 | 1798 | hsa-miR-6812-3p |
| hsa_circTADA2A_007 | hsa_circ_0106711 | 1798 | hsa-miR-6825-5p |
| hsa_circTADA2A_007 | hsa_circ_0106711 | 1798 | hsa-miR-6844 |
| hsa_circTADA2A_007 | hsa_circ_0106711 | 1798 | hsa-miR-6859-5p |
| hsa_circTADA2A_007 | hsa_circ_0106711 | 1798 | hsa-miR-6875-5p |
| hsa_circTADA2A_007 | hsa_circ_0106711 | 1798 | hsa-miR-6885-3p |
| hsa_circTADA2A_007 | hsa_circ_0106711 | 1798 | hsa-miR-7114-5p |
| hsa_circTADA2A_007 | hsa_circ_0106711 | 1798 | hsa-miR-765 |
| hsa_circTADA2A_007 | hsa_circ_0106711 | 1798 | hsa-miR-7703 |
| hsa_circTADA2A_007 | hsa_circ_0106711 | 1798 | hsa-miR-8082 |
| hsa_circTADA2A_007 | hsa_circ_0106711 | 1798 | hsa-miR-9-5p |
| hsa_circTADA2A_007 | hsa_circ_0106711 | 1798 | hsa-miR-941 |
|  |  |  |  |
