# Supplementary figures and images for: CircTADA2A suppresses the progression of colorectal cancer via miR-374a-3p/KLF14 axis
Source: J Exp Clin Cancer Res. 2020 Aug 15;39:160. doi: 10.1186/s13046-020-01642-7 (PMC7429896; doi:10.1186/s13046-020-01642-7)

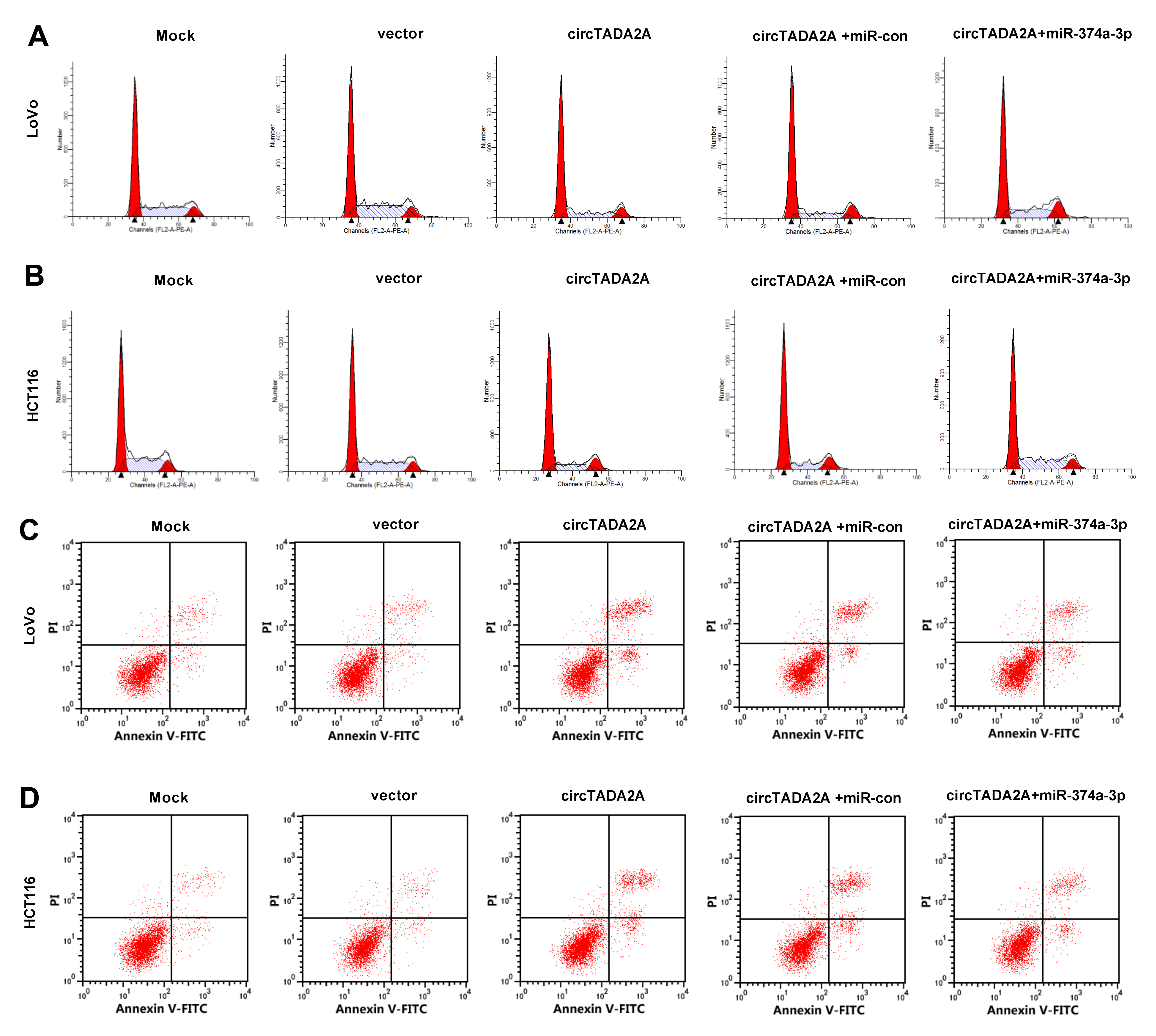

Supplement: Supplementary file 3 — Additional file 3: Supplementary Figure 1. The images of cell cycle and apoptosis in Fig. 5 [file 13046_2020_1642_MOESM3_ESM.tif]

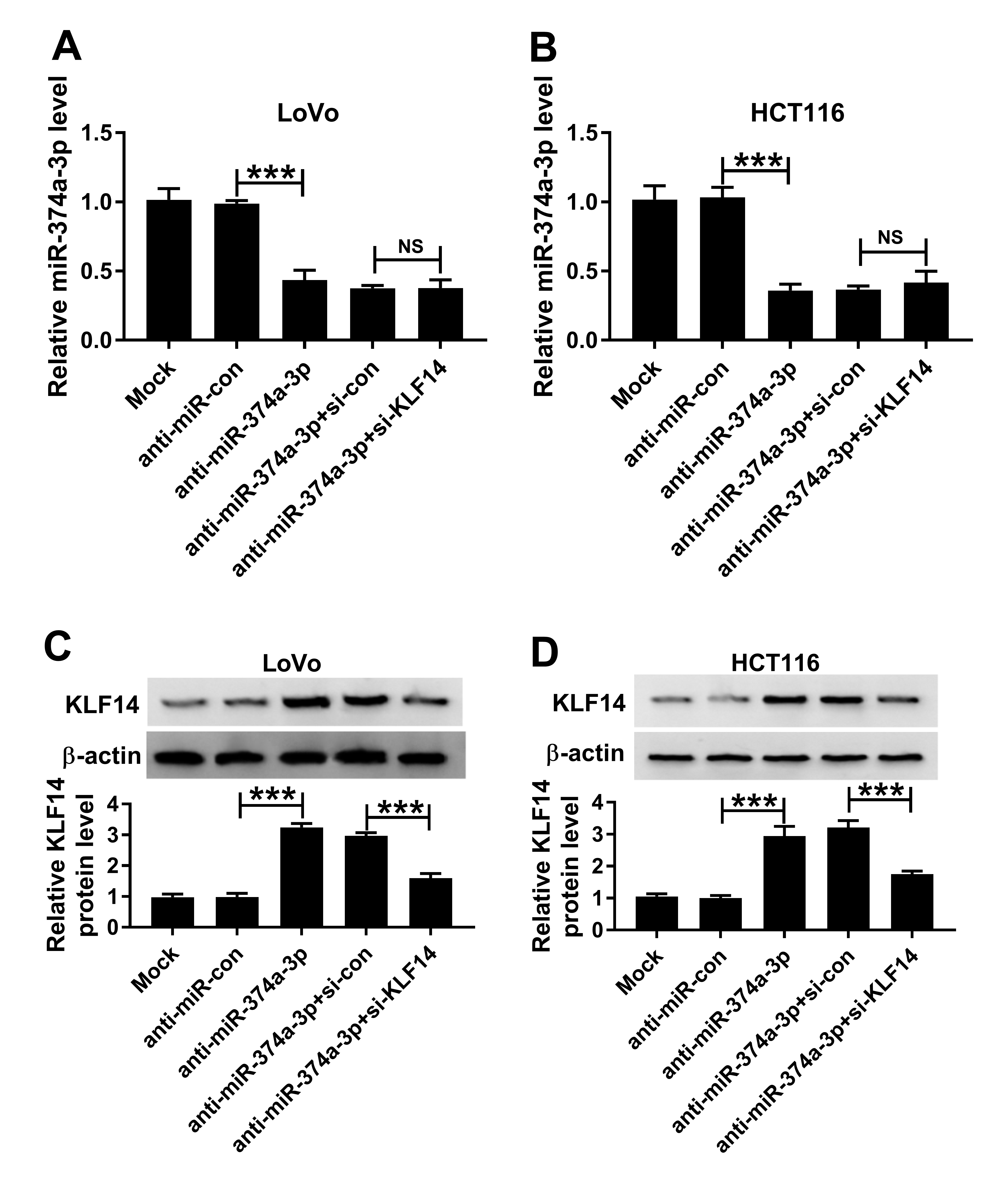

Supplement: Supplementary file 4 — Additional file 4: Supplementary Figure 2. The expression of miR-374a-3p and KLF14 in CRC cells under similar condition in Fig. 7. (A and B) The expression of miR-374a-3p was examined in CRC cells transfected with anti-miR-con, anti-miR-374a-3p, anti-miR-374a-3p + si-con or anti-miR-374a-3p + si-KLF14 by qRT-PCR. (C and D) Western blot assay was used to detect the protein level of KLF14 in CRC cells transfected with anti-miR-con, anti-miR-374a-3p, anti-miR-374a-3p + si-con or anti-miR-374a-3p + si-KLF14. ***P < 0.001. [file 13046_2020_1642_MOESM4_ESM.tif]
